# Prioritization of cancer driver gene with prize-collecting steiner tree by introducing an edge weighted strategy in the personalized gene interaction network

**DOI:** 10.1186/s12859-022-04802-y

**Published:** 2022-08-16

**Authors:** Shao-Wu Zhang, Zhen-Nan Wang, Yan Li, Wei-Feng Guo

**Affiliations:** 1 Key Laboratory of Information Fusion Technology of Ministry of Education, School of Automation, Northwestern Polytechnical University, Xi’an, 710072 China; 2 School of Electrical Engineering, Zhengzhou University, Zhengzhou, 450001 China

**Keywords:** Driver gene, Personalized cancer, Gene interaction network, Prize-collecting Steiner tree

## Abstract

**Background:**

Cancer is a heterogeneous disease in which tumor genes cooperate as well as adapt and evolve to the changing conditions for individual patients. It is a meaningful task to discover the personalized cancer driver genes that can provide diagnosis and target drug for individual patients. However, most of existing methods mainly ranks potential personalized cancer driver genes by considering the patient-specific nodes information on the gene/protein interaction network. These methods ignore the personalized edge weight information in gene interaction network, leading to false positive results.

**Results:**

In this work, we presented a novel algorithm (called PDGPCS) to predict the Personalized cancer Driver Genes based on the Prize-Collecting Steiner tree model by considering the personalized edge weight information. PDGPCS first constructs the personalized weighted gene interaction network by integrating the personalized gene expression data and prior known gene/protein interaction network knowledge. Then the gene mutation data and pathway data are integrated to quantify the impact of each mutant gene on every dysregulated pathway with the prize-collecting Steiner tree model. Finally, according to the mutant gene’s aggregated impact score on all dysregulated pathways, the mutant genes are ranked for prioritizing the personalized cancer driver genes. Experimental results on four TCGA cancer datasets show that PDGPCS has better performance than other personalized driver gene prediction methods. In addition, we verified that the personalized edge weight of gene interaction network can improve the prediction performance.

**Conclusions:**

PDGPCS can more accurately identify the personalized driver genes and takes a step further toward personalized medicine and treatment. The source code of PDGPCS can be freely downloaded from https://github.com/NWPU-903PR/PDGPCS.

**Supplementary Information:**

The online version contains supplementary material available at 10.1186/s12859-022-04802-y.

## Background

Cancer is an evolutionary process in which normal cells accumulate various kinds of epigenomic alterations and genomic variations, such as chromosomal aberrations, single nucleotide variations [1]. Some of these alterations/ variations confer growth and positive selection advantage to the mutant cells, resulting in intensive proliferation and tumors [2]. During tumor progression, majority of the altered genes are passengers that do not contribute to the oncogenic process, while a small part of genomic and transcriptomic altered genes is known as cancer driver genes that modify transcriptional programs [3]. It is a challenge to distinguish the driver mutations that promote the cancer development from those passenger mutations without selective advantages [4]. Recently, many computational methods have been proposed to identify driver genes from cancer genomics data. According to the significant features, these computational methods of identifying cancer driver genes can be cataloged into two groups: methods in large cohorts, and methods for individual patients. (1) Methods of identifying cancer driver gene in large cohorts. These computational methods mainly integrate multi-omics data from large cohorts and the topology properties of the gene–gene association (or protein–protein interaction) networks from the large-scale mixed experimental data rather than cell type specific, tissue specific or condition specific data [5–11]. However, due to the limited number of personalized sample information (e.g., the personalized omics data), it is difficult to apply these methods for effectively identifying the individual cancer patient-specific driver genes. (2) Methods of identifying cancer driver gene for individual patients. With the rapid development of high-throughput biological molecule screening, the emergence of systems biology has raised the possibility of exploring the personalized cancer driver genes from a network perspective for individual patient treatment. Thus, some computational methods of Prodigy [1], SCS [12], DawnRank [13], driveR [14] and IMCDriver [15] have been developed for identifying the personalized cancer driver genes from multi-dimensional genomic data. However, most of existing computational methods mainly rank the potential personalized cancer driver genes by considering the patient-specific node information on the gene–gene association (or protein–protein interaction) networks, which ignore the personalized edge weight information. Therefore, these existing methods may miss the important driver genes of individual cancer patients.

To address these methodological limitations, here we proposed an effective method (called PDGPCS) to identify the Personalized cancer Driver Genes with Prize-Collecting Steiner tree by considering the personalized edge weight information in gene interaction network to assess the impact of mutant genes. In detail, PDGPCS firstly used a paired single sample network construction approach (called paired-SSN) [3] to construct the personalized weighted gene interaction network for capturing the co-expression difference between normal state and tumor state on the gene/protein interaction network. The personalized weighted gene interaction network is a graph, in which the nodes represent the genes, and edges denote the significant co-expression difference between normal state and tumor state. Then, on the personalized weighted gene interaction network, we identified the differentially expressed genes, and defined the pathways with significantly enrich differentially expressed genes as dysregulated pathways, and also selected the personalized co-mutated genes as key mutant genes of an individual patient by adopting the random walk with restart (RWR) strategy [12] and hub genes selection strategy. Finally, a mutation-dysregulation network was constructed by using a mutant gene and a dysregulated pathway. In view of the individual differences, we used the gene expression data of individual patients to measure the edge weight of personalized weight gene interaction network, and then adopted the Prize-Collecting Steiner Tree (PCST) model to quantify the impact of mutant genes on the dysregulated pathway, so as to predict the individual cancer driver genes. PDGPCS also allows us to apply the Condorcet method to determine the ranking of genes in a patient population, and top-50 ranks candidates are selected as the driver mutations for the patient population. We evaluated the performance of PDGPCS in predicting the personalized cancer driver genes on four different benchmark datasets of Bladder Urothelial Carcinoma (BLCA), Colon adenocarcinoma (COAD), Head and Neck squamous cell carcinoma (HNSC), Breast invasive carcinoma (BRCA).

## Results and discussion

### Overview of the method

**Fig. 1:**
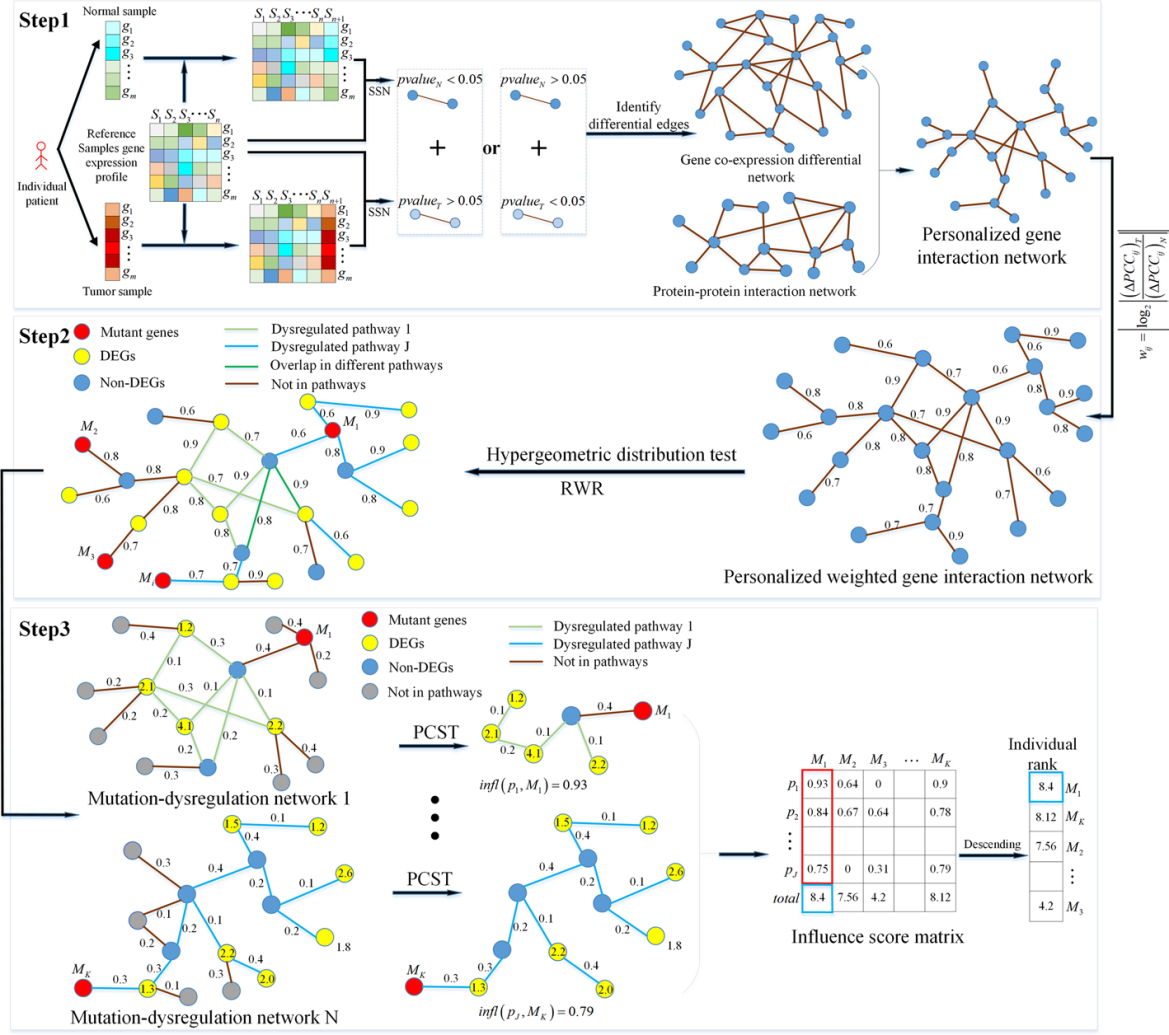
Schematic diagram of PDGPCS for prioritizing the personalized driver genes

The algorithm of PDGPCS consists of three steps, depicted in Fig. 1. In step 1, we used the paired-SSN method [3] to construct the personalized weighted gene interaction network from gene expression data of individual patient. In step 2, from the gene expression data of tumor and normal samples of a patient, we identified the differentially expressed genes by fixing a threshold of fold change, and pick out the dysregulated pathway with hypergeometric distribution test. Then, we adopted the random walk with restart (RWR) algorithm to screen the key mutant genes. In step 3, for a mutant gene and a dysregulated pathway, we built its weighted mutation-dysregulation subnetwork, and then used the Prize-Collecting Steiner tree model to quantify the impact score of mutant gene. Finally, we ranked all mutant genes with their aggregated impact score on all dysregulated pathways to prioritize the personalized cancer driver genes.

We should note that our PDGPCS is a method which can predict the personalized driver genes of individual patients. Because the known gold-standard of personalized driver genes is not available, we cannot directly evaluate the performance of PDGPCS in terms of predicting personalized driver genes directly. Here, we used the common strategies adopted in current personalized cancer driver prediction methods [1] to evaluate the performance of PDGPCS. That is, we obtained the prediction results of PDGPCS in the entire cohorts by using Condorcet voting method to integrate the results of different individual patients, and thus evaluated our PDGPCS’s performance with the gold-standard of cancer driver genes for the entire cohorts (*i.e*., Cancer Genes census [16]). Here, we selected top 50 ranked mutant genes in the population as the candidate driver genes for assessing the performance of our PDGPCS.

### Performance of PDGPCS for identifying cancer driver genes

To access the performance of our PDGPCS, we compared PDGPCS with other state-of-the-art methods of PRODIGY [1], SCS [12] and three centrality measures (i.e., Degree, Betweenness and Closeness) on the genomics data of individual patients from four TCGA cohorts, such as BLCA, BRCA, COAD and HNSC (19, 100, 27 and 43 patients, respectively). For PRODIGY and SCS method, we used the same multi- genomics data of individual patients as our PDGPCS including gene mutation data (i.e., SNV and CNV), gene expression data (tumor and normal data), and protein interaction network. In addition, for all mutant genes, the same screening strategy (i.e., RWR and hub genes selection strategy) to select the candidate mutant genes as our PDGPCS was used for PRODIGY method.

**Fig. 2:**
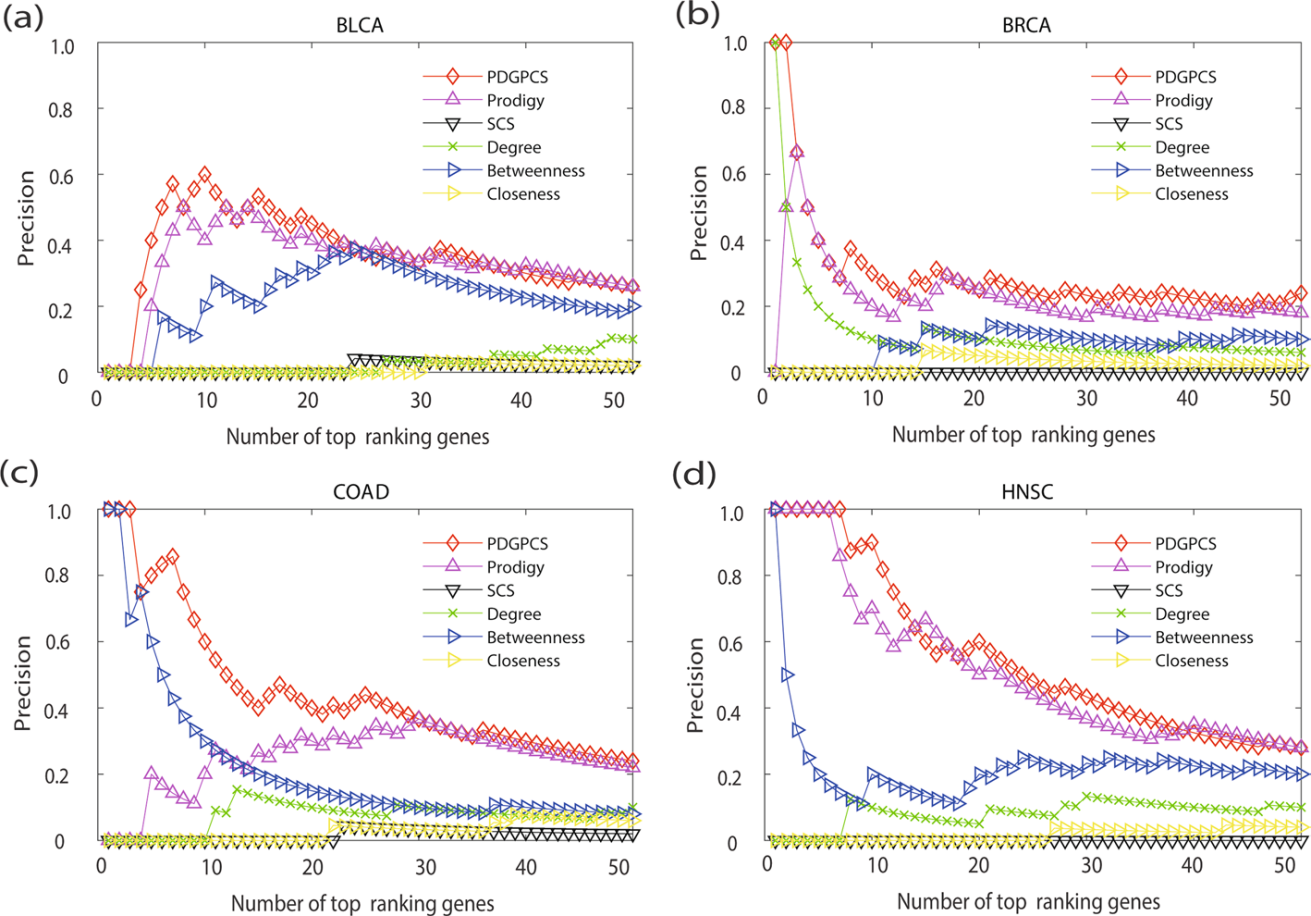
Precision of PDGPCS, PRODIGY, SCS and three centrality measures methods for predicting the driver genes on BLCA, BRCA, COAD, and HNSC cancers. **a **BLCA, **b** BRCA, **c** COAD, and **d** HNSC

**Fig. 3:**
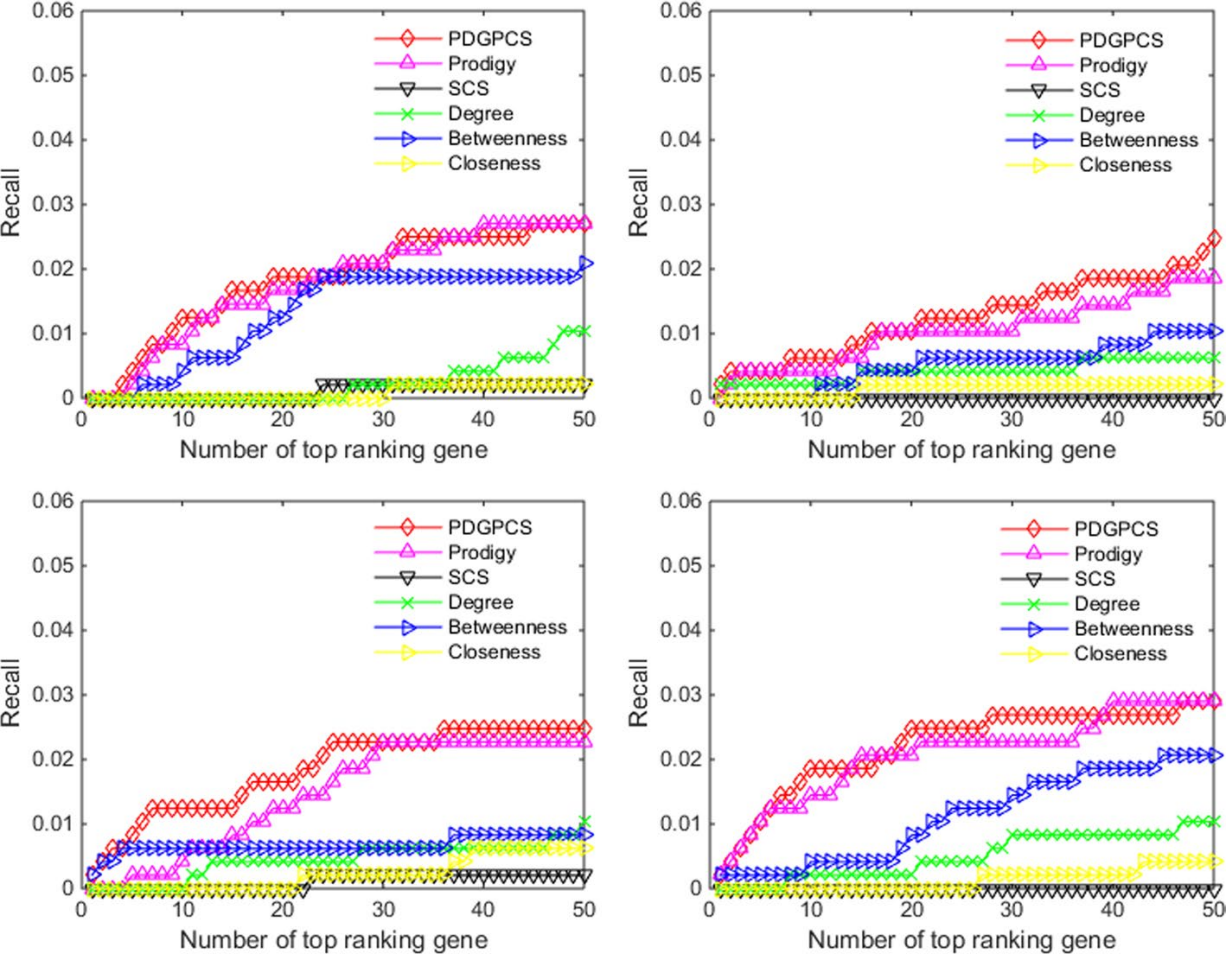
Recall of PDGPCS, PRODIGY, SCS and three centrality measures methods for predicting the driver genes on BLCA, BRCA, COAD, and HNSC cancers

**Fig. 4:**
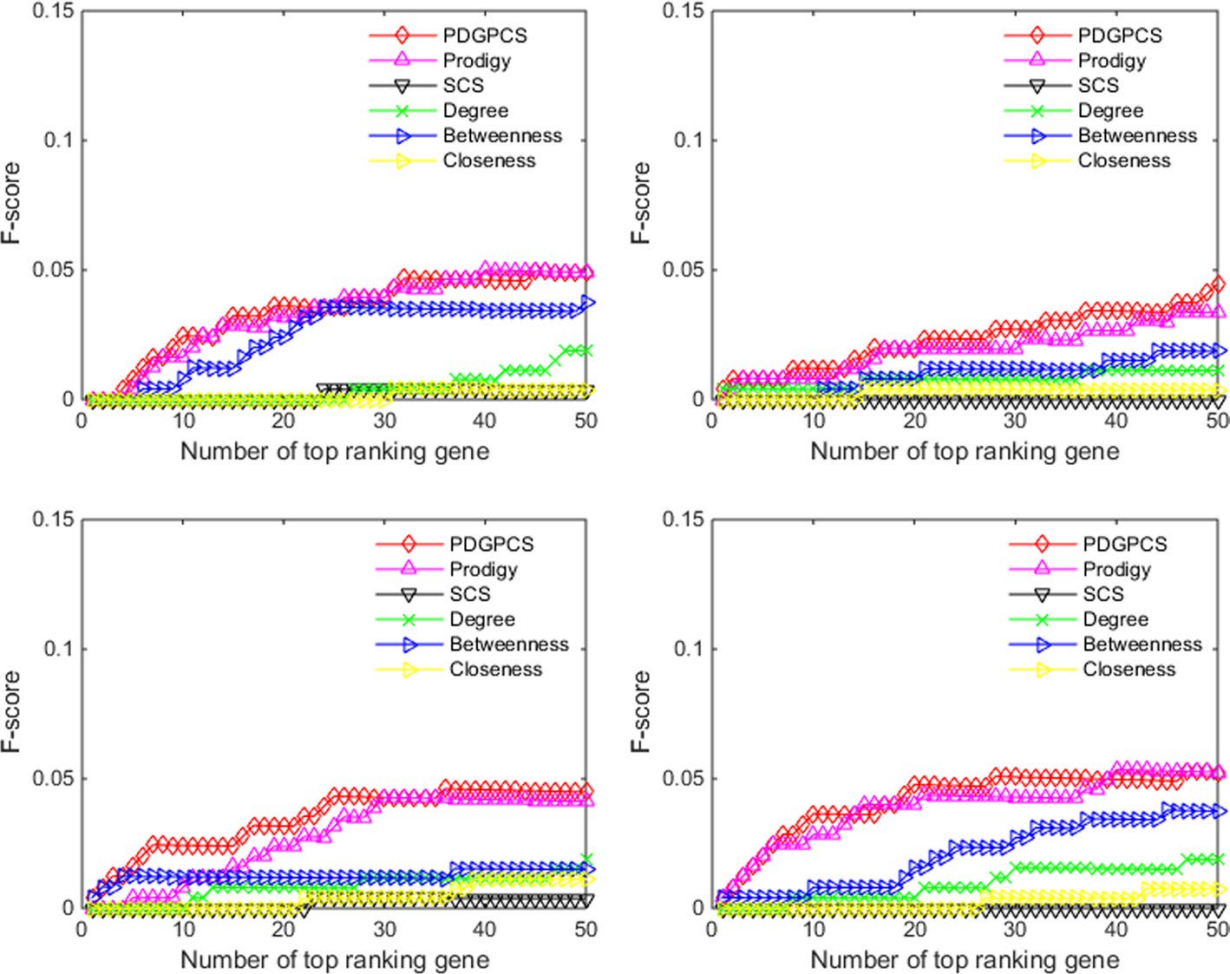
F-score of PDGPCS, PRODIGY, SCS and three centrality measures methods for predicting the driver genes on BLCA, BRCA, COAD, and HNSC cancers

From the prediction results of PDGPCS, PRODIGY, SCS and three centrality measures methods, we selected top 50 ranking genes as the cancer driver genes. Figs. 2, 3, 4 showed the precision, recall and F1-score of our PDGPCS, PRODIGY and three centrality measures methods on BLCA, BRCA, COAD and HNSC cancers, respectively. The ranking genes for PDGPCS, PRODIGY and three centrality measures methods on BLCA, BRCA, COAD and HNSC cancers were added in Additional file 2. From the results of Figs. 2, 3, 4, we can see that the values of precision, recall and F1 are consistent for different method. Furthermore, we can see that PDGPCS has better prediction performance than other methods on BLCA, BRCA, COAD and HNSC cancers. For example, in Fig. 2 if we selected top 50 ranking gene as the cancer driver genes, then the precision of our PDGPCS on BRCA cancer is 0.24, which is 0.06, 0.24, 0.18, 0.14 and 0.22 higher than that of PRODIGY, SCS, Degree, Betweenness and Closeness, respectively.

Furthermore, we also calculated the results of PDGPCS when choosing the KEGG pathways source dataset [17] and Reactome pathway source dataset, and compared these results with other methods. The precision, recall and F-score of these methods with Reactome pathways on four cancers were shown in Additional file 1: Fig. S1. From Additional file 1: Fig. S1, we can see that the performance of PDGPCS is better than PRODIGY [1], SCS [12] and three centrality measures (i.e., Degree, Betweenness and Closeness) no regardless of pathway datasets.

Recently some newer methods such as IMCDriver [18] has been proposed to predict personalized driver genes. However, these methods are the supervised learning methods, which need the information of known driver genes in benchmark data. Therefore, it is not improper to compare our PDGPCS with these supervised learning methods in terms of the precision results. Nevertheless, we compared our PDGPCS with IMCDriver in predicting cancer biomarker genes with significant survival analysis in the following section of “survival analysis for PDGPCS”.

### Robustness of PDGPCS for identifying the driver genes

To demonstrate the robustness of our PDGPCS to the number of reference samples on an individual patient, we calculated the proportion of non-significant edges when selecting a certain proportion of reference samples (i.e., normal samples) to construct the corresponding gene interaction network. The computational details are shown as below: (1) Firstly, we randomly selected a certain proportion of reference samples (i.e., normal samples) to construct the corresponding null distribution of gene interaction network (e.g., 30%), and this procedure was repeated by 100 times. (2) Then, we obtained the null distribution for the weights of all edges. (3) Finally, we calculated *P*-value of each edge that demonstrates whether the edge weight of the original personalized gene interaction network is significantly different from the null distribution.

**Fig. 5:**
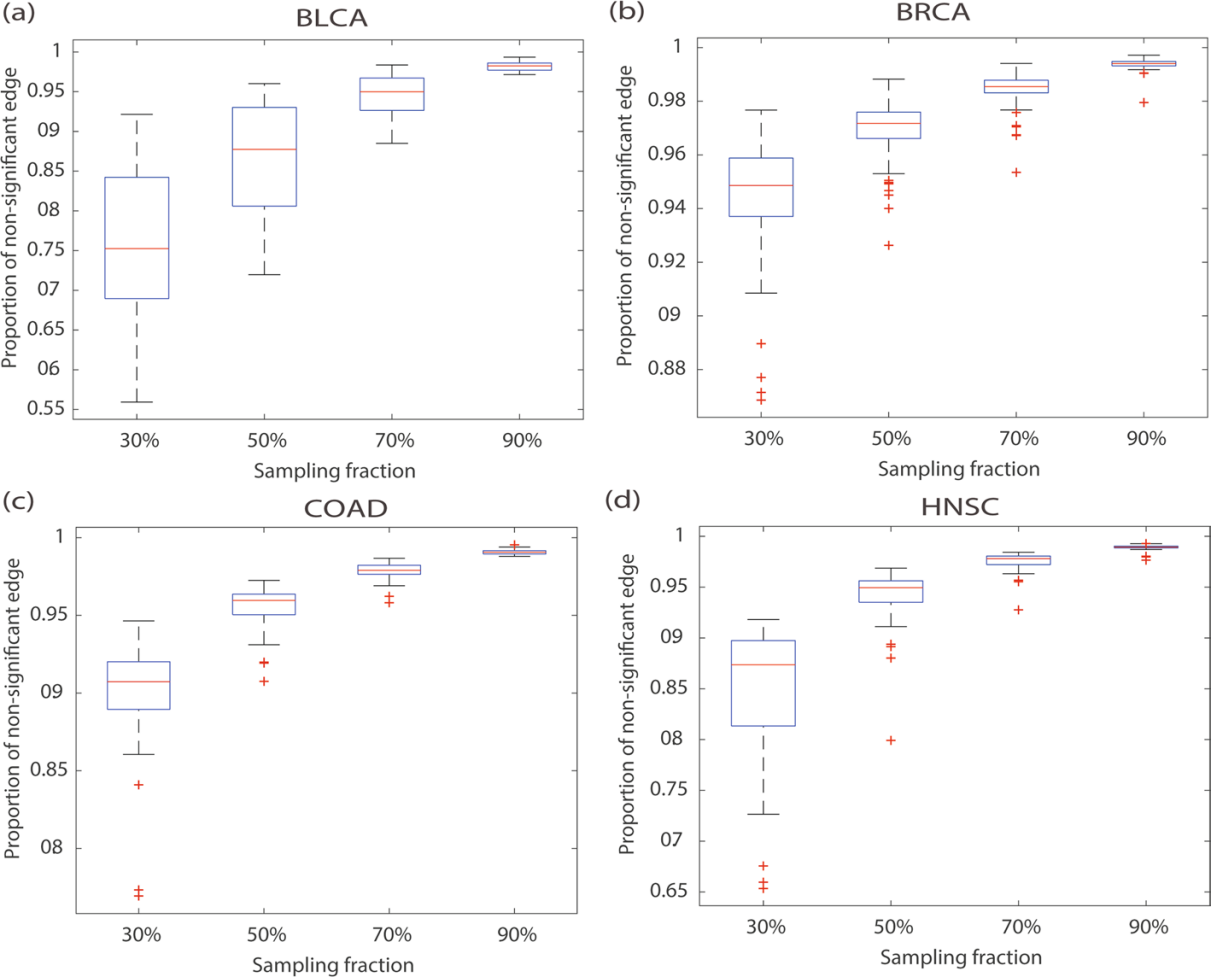
Robustness of PDGPCS with different reference sample numbers for BLCA, BRCA, COAD and HNSC. **a** BLCA, **b** BRCA, **c** COAD, **d** HNSC

We considered edges with *P*-value larger than 0.05 as non-significant edges in the personalized gene interaction network. Figure 5 showed the error bar of robustness value of all patients under 10%, 30%, 50%, 70% and 90% of all reference samples on four cancers, respectively. As shown in Fig. 5, we can see that as the number of reference samples increases, the robustness value gradually increases. Furthermore, when the number of samples is large (≥ 70%), the performance of PDGPCS tends to be stable.

### The weighted strategy in personalized gene interaction network improving the performance of PDGPCS

**Fig. 6:**
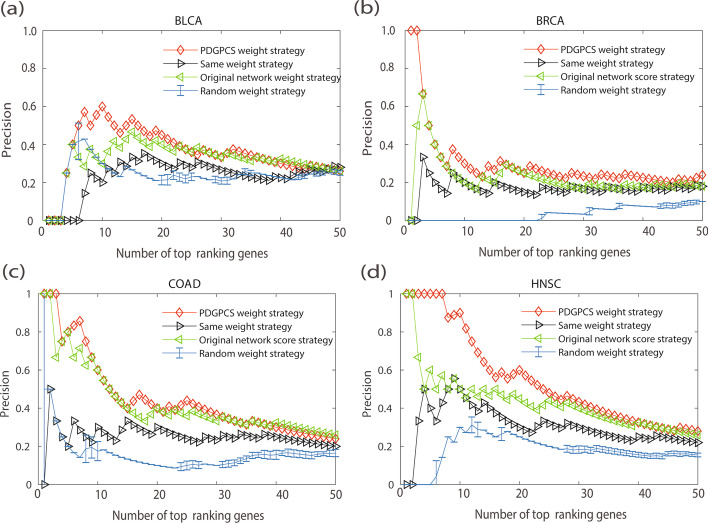
Precision of PDGPCS with the PDGPCS weight strategy, random weight strategy, same weight strategy and original network weight strategy for predicting cancer driver genes on BLCA, BRCA, COAD and HNSC cancers, respectively. For random weighted strategy, the precision is the error bar result of 10 runs. **a **BLCA, **b **BRCA, **c **COAD, and **d **HNSC

**Fig. 7:**
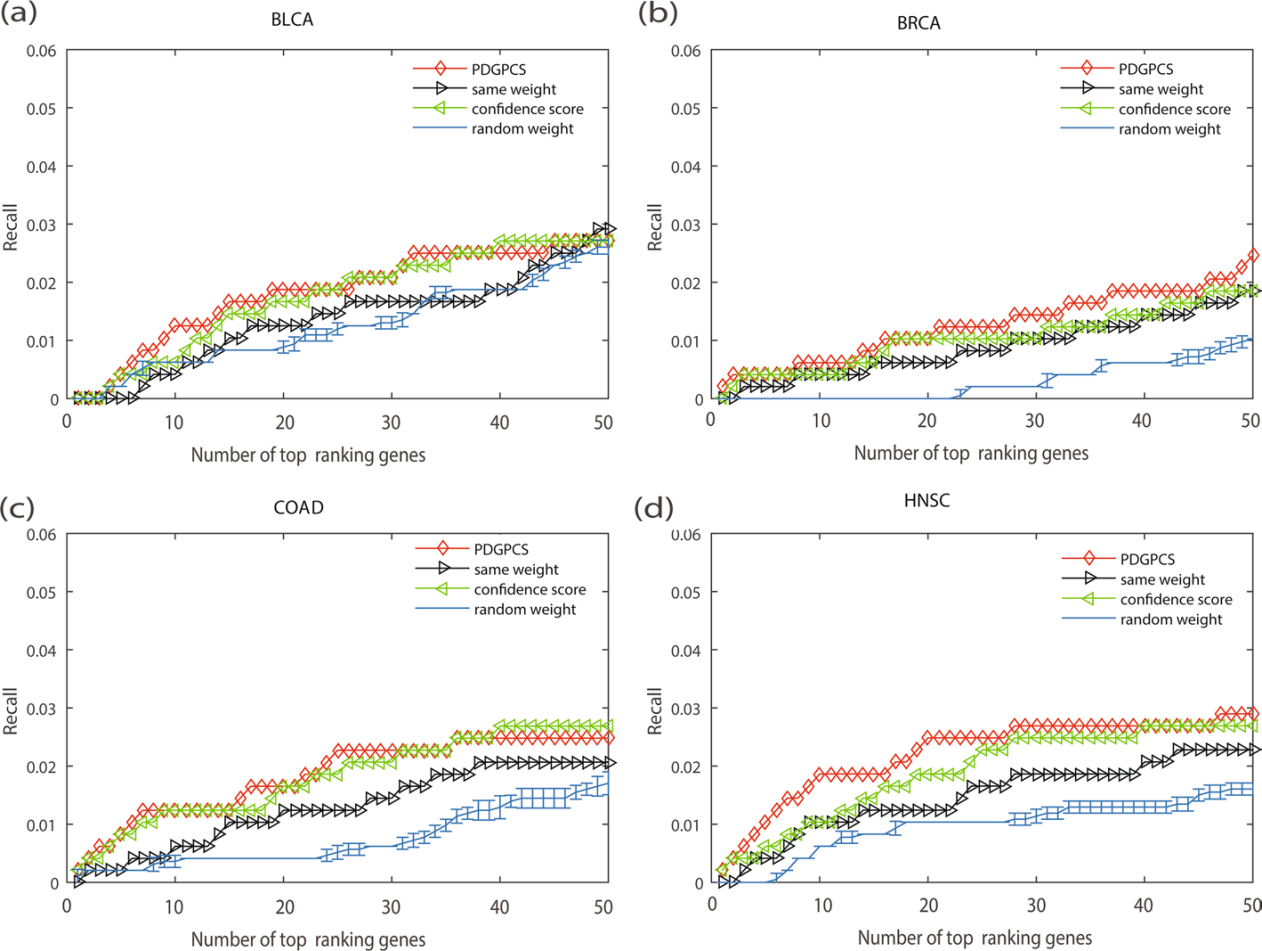
Recall of PDGPCS with the PDGPCS weight strategy, random weight strategy, same weight strategy and original network weight strategy for predicting cancer driver genes on BLCA, BRCA, COAD and HNSC cancers, respectively. For random weighted strategy, the precision is the error bar result of 10 runs. **a** BLCA, **b** BRCA, **c** COAD, and **d **HNSC

**Fig. 8:**
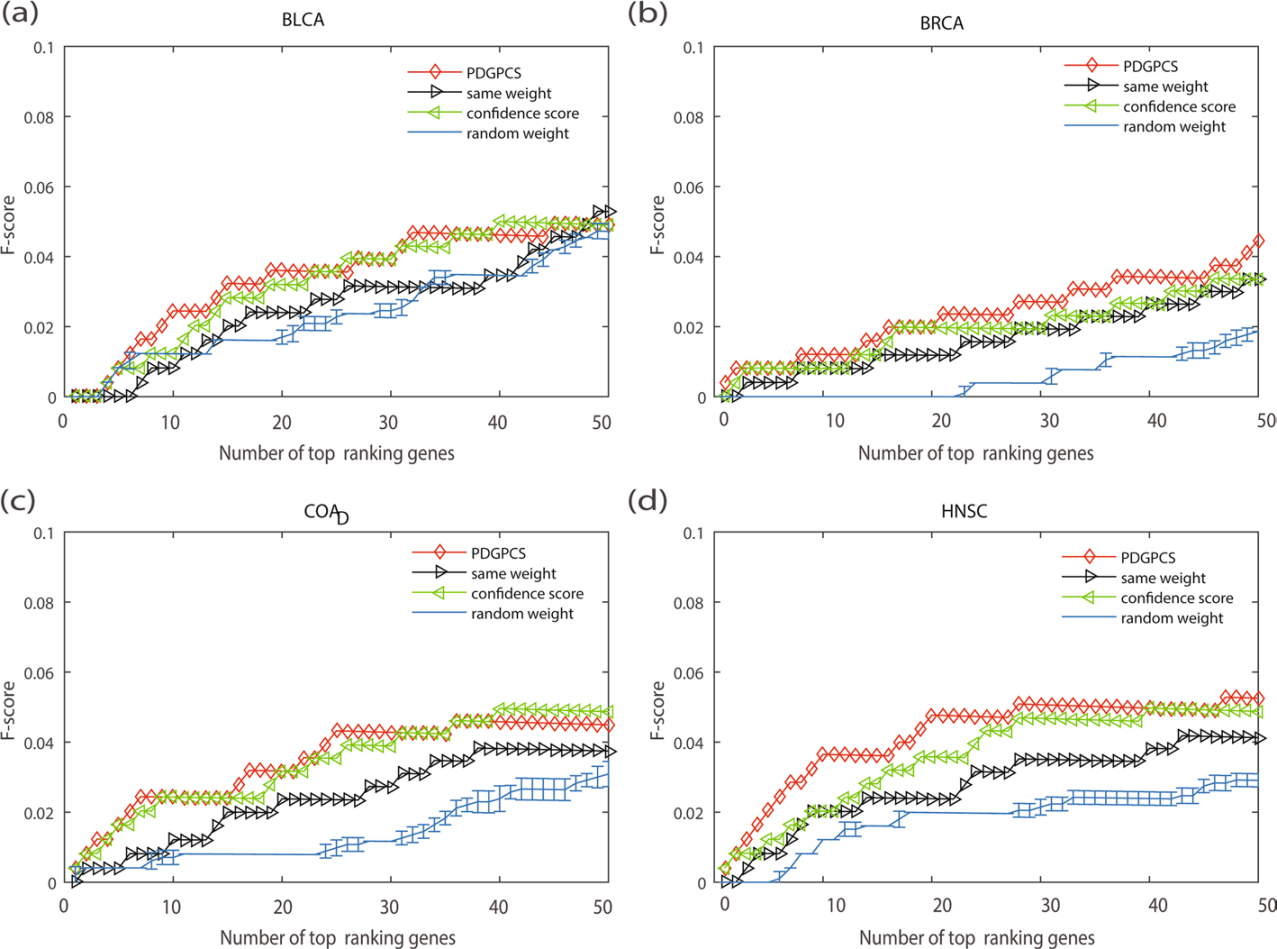
F-score of PDGPCS with the PDGPCS weight strategy, random weight strategy, same weight strategy and original network weight strategy for predicting cancer driver genes on BLCA, BRCA, COAD and HNSC cancers, respectively. For random weighted strategy, the precision is the error bar result of 10 runs. **a** BLCA, **b **BRCA, **c **COAD, and **d **HNSC

The main advantage of our PDGPCS is that we used the gene expression data of individual patients to assign the personalized edge weights (or costs) in gene interaction network, and this weighted strategy is called as the PDGPCS weight strategy. The personalized edge weights in gene interaction network reflects the gene interactions with significant co-expression differences between normal and tumor samples of an individual patient. In order to verify the effectiveness of this strategy, we randomly assigned the weights (in the range of 0–1) to the edges in gene interaction network, and implemented our PDGPCS on BLCA, BRCA, COAD and HNSC cancer datasets, and this weighted strategy is called as the random weight strategy. After repeating 10 times, we compared the results of gene expression weighted strategy and random weighted strategy in a patient population. We also assigned the weight of all edges in gene interaction network to 1, and implementing PDGPCS on four cancer datasets, and this weighted strategy is called as the same weight strategy. In fact, here we used the physical interactions with confidence score > 0.7 that were validated experimentally from other curated databases in the reference gene/protein interaction network data [1]. Therefore, we also assigned the weights of edges in gene interaction network as its original network score, and implementing PDGPCS on four cancer datasets, and this weighted strategy is called as the original network weight strategy. The comparison results were shown in Figs. 6, 7 and 8.

As shown in Figs. 6, 7 and 8, we can see that the precision, recall and F-score of PDGPCS weighted strategy (i.e., gene expression weighted strategy) were significantly higher than those of random weighted strategy on these four cancer datasets. For example, if we selected top 50 ranking gene as the cancer driver genes, then the precision of gene expression weighted strategy for BRCA is 0.24, which is 0.06, 0.06 and 0.14 higher than that of original network score strategy, same weight strategy and random weight strategy, respectively. Therefore, the results in Figs. 6, 7 and 8 showed that it is effective by using the PDGPCS weight strategy of individual cancer patients to measure the personalized edge weights in gene interaction network.

### The paired samples approach improving the performance of PDGPCS

**Fig. 9:**
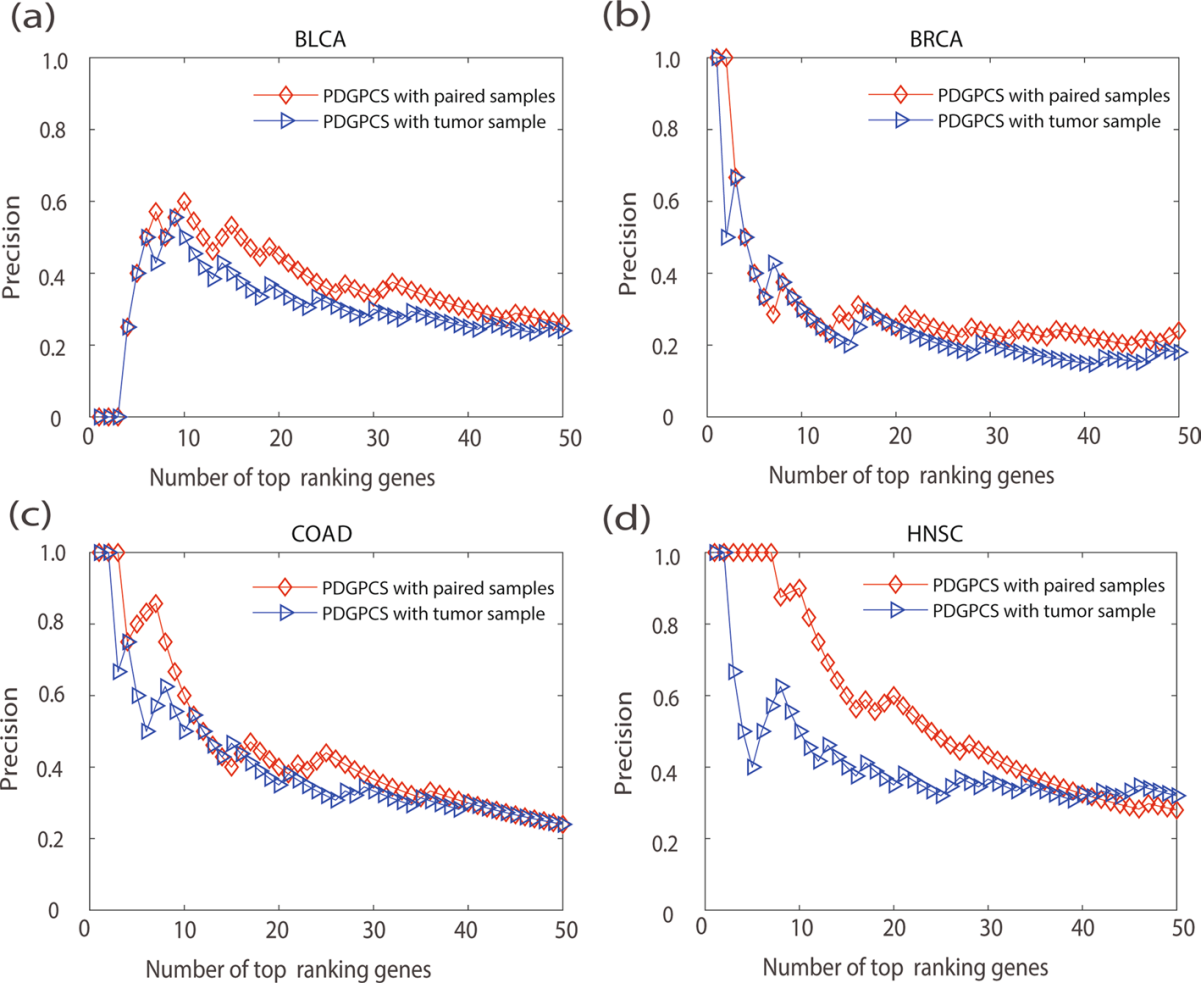
Precision of PDGPCS with tumor sample alone trick and the paired samples approach to predict the potential cancer driver genes on BLCA, BRCA, COAD and HNSC cancers,respectively. **a **BLCA, **b **BRCA, **c **COAD, and **d **HNSC

**Fig. 10:**
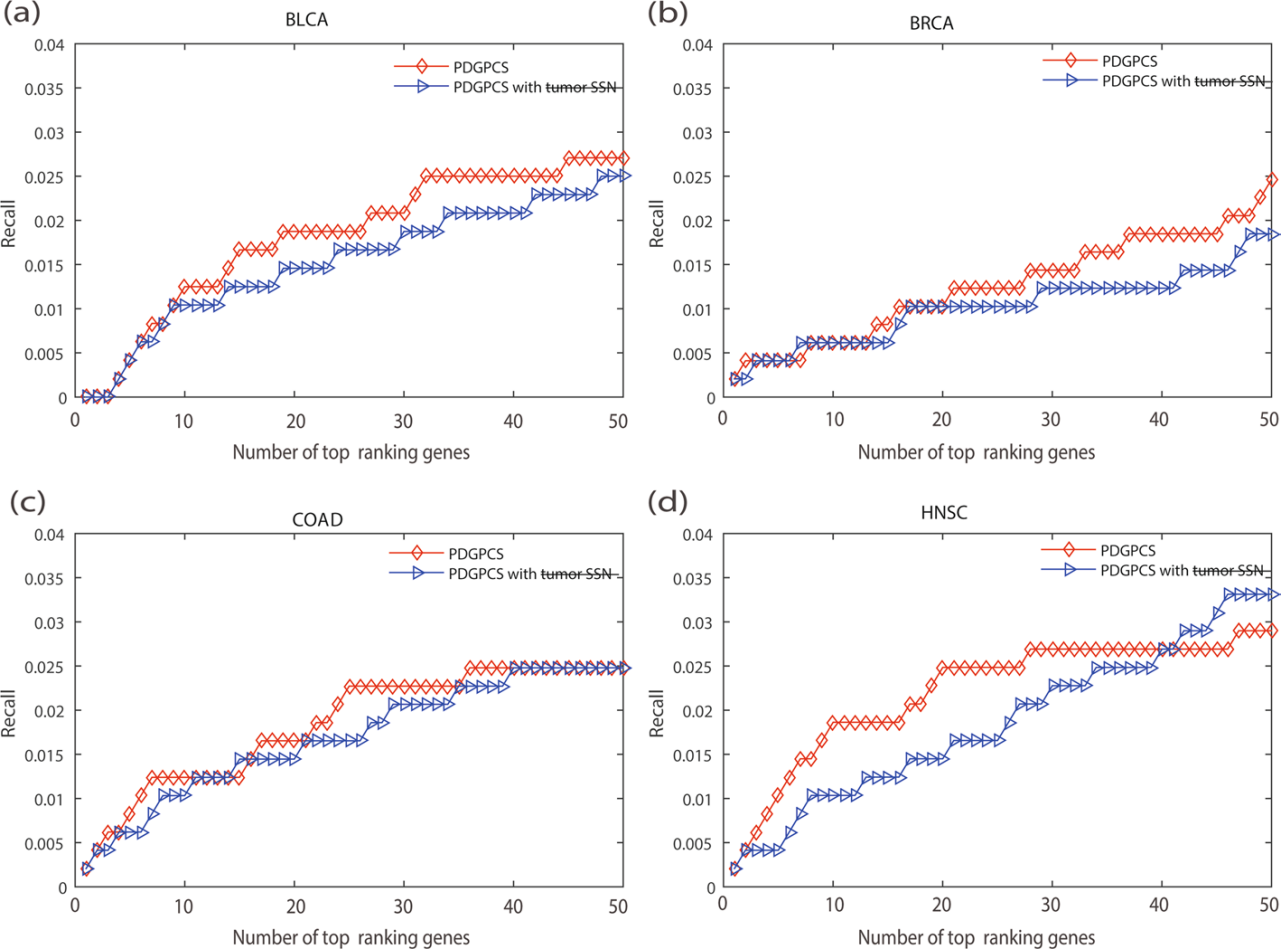
Recall of PDGPCS with tumor sample alone trick and the paired samples approach to predict the potential cancer driver genes on BLCA, BRCA, COAD and HNSC cancers, respectively. **a** BLCA, **b** BRCA, **c **COAD, and **d **HNSC

**Fig. 11:**
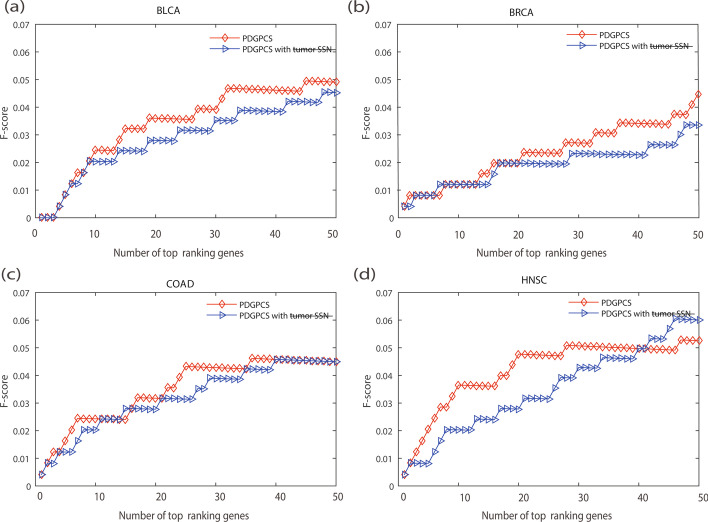
F-score of PDGPCS with tumor sample alone trick and the paired samples approach to predict the potential cancer driver genes on BLCA, BRCA, COAD and HNSC cancers, respectively. **a** BLCA, **b **BRCA, **c **COAD, and **d **HNSC

A characteristic of our PDGPCS is that we used paired samples (i.e., tumor sample data and normal sample data) of each individual patient to construct the personalized weight gene interaction network. To verify the effect of the paired samples approach on PDGPCS, we compared the results of PDGPCS with tumor sample alone and PDGPCS with paired samples. To obtain the results of PDGPCS with the tumor sample, for an individual patient, we firstly removed the normal sample of this patient and used all other normal samples to construct the reference network in step 1 of PDGPCS. Then, we only used the tumor sample of this patient to construct personalized weighted gene interaction network by using the SSN method for this individual patient. The weight of edge $e_{ij}$ is calculated as $w_{ij} = \left| {\left( {\Delta PCC_{ij} } \right)_{Tumor} } \right|$. Finally, on BLCA, BRCA, COAD and HNSC cancer datasets, we implemented step 2 and step 3 in PDGPCS to obtain the potential cancer driver genes.The comparison results were shown in terms of the precision, recall and F-score metric for PDGPCS with paired samples approach and single tumor sample in Figs. 9, 10 and 11.

As shown in Figs. 9, 10 and 11, we can see that the precision, recall and F-score of PDGPCS with the paired samples approach is higher than those of PDGPCS with tumor sample alone trick on these four cancer datasets. The results in Figs. 9, 10 and 11 demonstrated that the paired samples approach can improve the performance of our PDGPCS. Furthermore, to let readers see the detailed results, in Tables 1, 2 and 3 we gave the overall results of average precision, recall and F-score among the top *k* (*k* = 1, 2,…, 50) ranking predicted driver genes for each type of cancer, and these results were also shown in Figs. 2, 3, 4, 6, 7, 8, 9, 10 and 11. The detailed results in terms of the precision, recall and F-score shown in the Figs. 2, 3, 4, 6, 7, 8, 9, 10 and 11 are also provided in Additional files 3, 4 and 5.

**Table 1:** Results of average precision of PDGPCS and other methods (or strategies) on BLCA, BRCA, COAD and HNSC cancers

Method/Strategy	BLCA	BRCA	COAD	HNSC
PDGPCS	0.3623	0.2968	0.4534	0.5512
PRODIGY	0.3311	0.2265	0.2479	0.5134
SCS	0.0152	0	0.0162	0
Degree	0.0262	0.1214	0.0735	0.0807
Betweenness	0.2260	0.0828	0.2198	0.2241
Closeness	0.0101	0.0250	0.0290	0.0163
Same weight	0.2330	0.1678	0.2545	0.3064
Original network weight	0.3160	0.2265	0.4292	0.4314
Random weight	0.2383	0.0358	0.1589	0.1802
Tumor sample	0.0077	0.0027	0.0235	0.0102

**Table 2:** Results of average Recall of PDGPCS and other methods (or strategies) on BLCA, BRCA, COAD and HNSC cancers

Method/Strategy	BLCA	BRCA	COAD	HNSC
PDGPCS	0.018	0.0126	0.0185	0.0221
PRODIGY	0.0177	0.0103	0.0145	0.0208
SCS	0.0011	0	0.001	0
Degree	0.0022	0.0041	0.0043	0.0050
Betweenness	0.01308	0.0051	0.0066	0.01118
Closeness	0.0008	0.0014	0.0022	0.0013
Same weight	0.0136	0.0087	0.0128	0.0148
Original network weight	0.0174	0.0103	0.0181	0.01908
Random weight	0.0125	0.0028	0.0073	0.0096
Tumor sample	0.01537	0.0102	0.0170	0.0186

**Table 3:** Results of F-scores of PDGPCS and other methods (or strategies) on BLCA, BRCA, COAD and HNSC cancers

Method/Strategy	BLCA	BRCA	COAD	HNSC
PDGPCS	0.0343	0.0236	0.0348	0.0418
PRODIGY	0.0332	0.0194	0.0272	0.0393
SCS	0.0020	0	0.0021	0
Degree	0.0041	0.0077	0.0081	0.0093
Betweenness	0.0245	0.0095	0.0125	0.0208
Closeness	0.00153	0.0027	0.00420	0.0024
Same weight	0.0255	0.0164	0.0241	0.0278
Original network weight	0.0327	0.0194	0.0341	0.0358
Random weight	0.0237	0.0053	0.0138	0.0181
Tumor sample	0.0288	0.0192	0.0320	0.0350

### Effects of using different strategies to screen the key mutant genes in PDGPCS

To identify the personalized driver genes, a key step in our PDGPCS method is the utility of RWR and hub genes selection strategies to screen the key mutant genes (i.e., step 2 in PDGPCS). In Tables 4, 5 and 6, we evaluated the effects of using the RWR strategy, hub genes selection strategy, and their combination for our PDGPCS in terms of average precision, recall and F-score among the top *k* (*k* = 1, 2,…, 50) ranking predicted driver genes for each type of cancer. From the results in Table 1, we can see that average precision, recall and F-score of the combination of two strategies is significantly higher than that of any one strategy and the strategy of using all mutant genes. For example, the average precision of combination strategy on BLCA is 0.3623, which is 0.0276, 0.1335 and 0.2056 higher than that of the RWR strategy alone, hub genes selection strategy alone and strategy of using all mutant genes, respectively. These results showed that the combination strategy of RWR and Hub gene selection can improve the performance of PDGPCS.

**Table 4:** Average precision of using different strategy to screen the key mutant genes in PDGPCS for BLCA, BRCA, COAD and HNSC cancers

Strategy	BLCA	BRCA	COAD	HNSC
RWR + Hub genes selection	0.3623	0.2968	0.4534	0.5512
RWR	0.3347	0.1678	0.4018	0.2701
Hub genes selection	0.2288	0.1398	0.2666	0.1570
Using all mutant genes	0.1567	0.0704	0.1013	0.0443

**Table 5:** Average recall of using different strategy to screen the key mutant genes in PDGPCS for BLCA, BRCA, COAD and HNSC cancers

Strategy	BLCA	BRCA	COAD	HNSC
RWR + Hub genes selection	0.0180	0.0126	0.0185	0.0221
RWR	0.0138	0.0069	0.0166	0.0112
Hub genes selection	0.0094	0.0058	0.0110	0.0065
Using all mutant genes	0.0065	0.0029	0.0042	0.0018

**Table 6:** Average F-score of using different strategy to screen the key mutant genes in PDGPCS for BLCA, BRCA, COAD and HNSC cancers

Strategy	BLCA	BRCA	COAD	HNSC
RWR + Hub genes selection	0.0343	0.0236	0.0348	0.0418
RWR	0.0266	0.013	0.0320	0.0215
Hub genes selection	0.0182	0.0111	0.0212	0.0125
Using all mutant genes	0.0124	0.0056	0.0080	0.0035

**Fig. 12:**
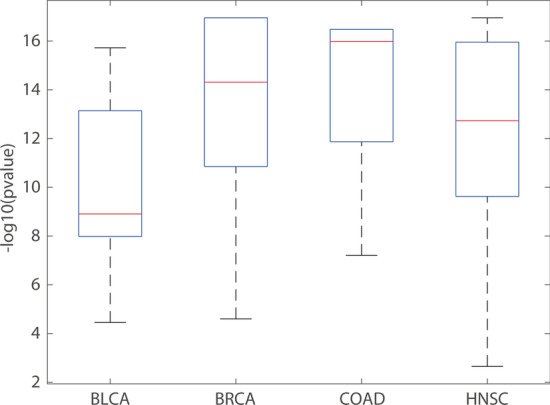
The enrichment of genes with a degree greater than the mean of degree in CGC dataset on BLCA, BRCA, COAD and HNSC cancers

In addition, in order to demonstrate the necessity of hub genes selection strategy, we counted the number of genes whose degree is greater than the mean of degree in the gene interaction network, and then we randomly selected the same number of genes in the gene interaction network (e.g., 1000 times) and calculated the null distribution of the number of these genes in CGC dataset for each individual patient. We calculated the enrichment of the number of genes whose degree is greater than the mean of degree in the gene interaction network in CGC dataset for each individual patient. The results were shown in Fig. 12.

As shown in Fig. 12, the genes with a degree greater than the mean of degree in the gene interaction network are significantly enriched in CGC dataset on four cancer datasets, indicating that the driver genes usually have a larger degree in the network. Thus, hub genes selection strategy is reasonable.

### The effects of parameter $\beta$ to identify the DEGs on PDGPCS

**Fig. 13:**
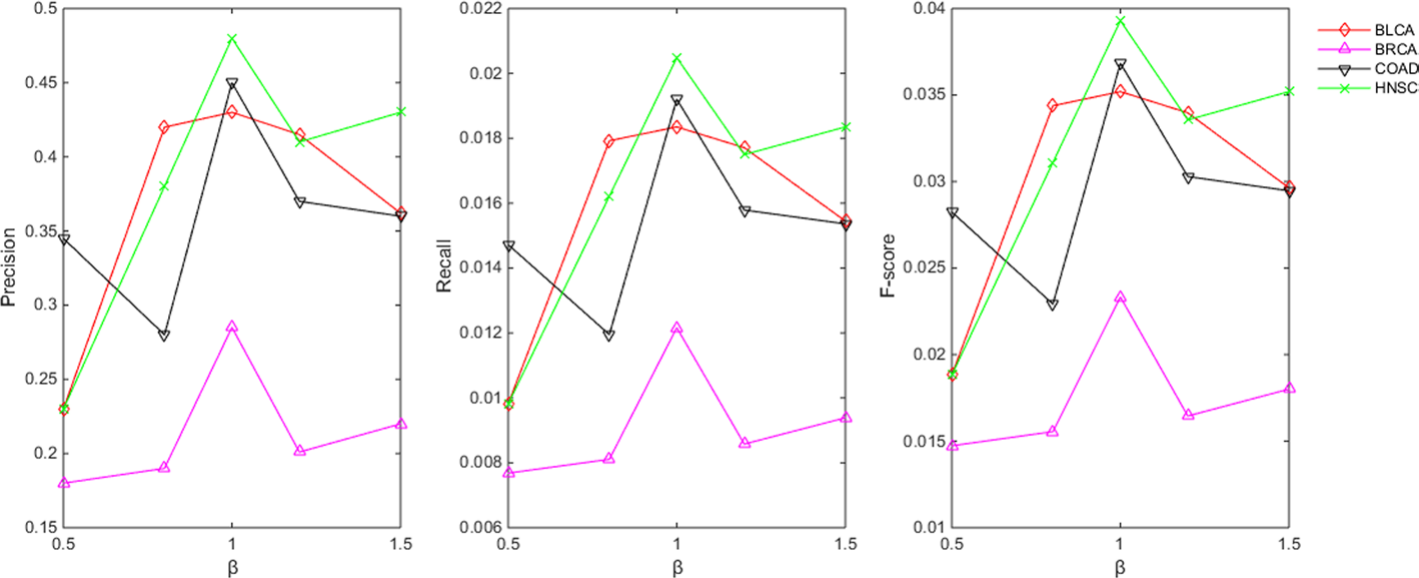
Average precision, recall and F-score of PDGPCS with different $\beta$ values among the top $k(k = 1,2, \ldots ,50)$ ranking predicted driver genes for BLCA, BRCA, COAD and HNSC cancers

Another key step in our PDGPCS is that we used the personalized DEGs to identify the dysregulated pathways of individual patients. In PDGPCS, we firstly calculated the fold change between the normal sample and the tumor sample of individual patients, and then selected the genes with $\left| {\log_{2} fold \, change} \right| > \beta$(here, we set $\beta = 1$) as the personalized DEGs. In fact, the threshold of $\left| {\log_{2} fold \, change} \right|$ to identify the DEGs was adopted in many previous research works [12, 19–21]. We also considered different values to evaluate the influence of the threshold $\beta$ on the precision of predicted driver genes. To assess the influence of parameter $\beta$ on our PDGPCS, we set $\beta$ with different values in range of $\left[ {0.5,{1}{\text{.5}}} \right]$. Average precision, recall and F-score of PDGPCS for four cancers with different $\beta$ values were shown on Fig. 13. As shown in Fig. 13, the parameter $\beta$ has a certain influence on the prediction performance of PDGPCS. When $\beta = 1$, PDGPCS has the highest average precision, recall and F-score for BLCA, BRCA, COAD and HNSC, thus we set $\beta = 1$ in this work.

### Enrichment analysis for PDGPCS

**Fig. 14:**
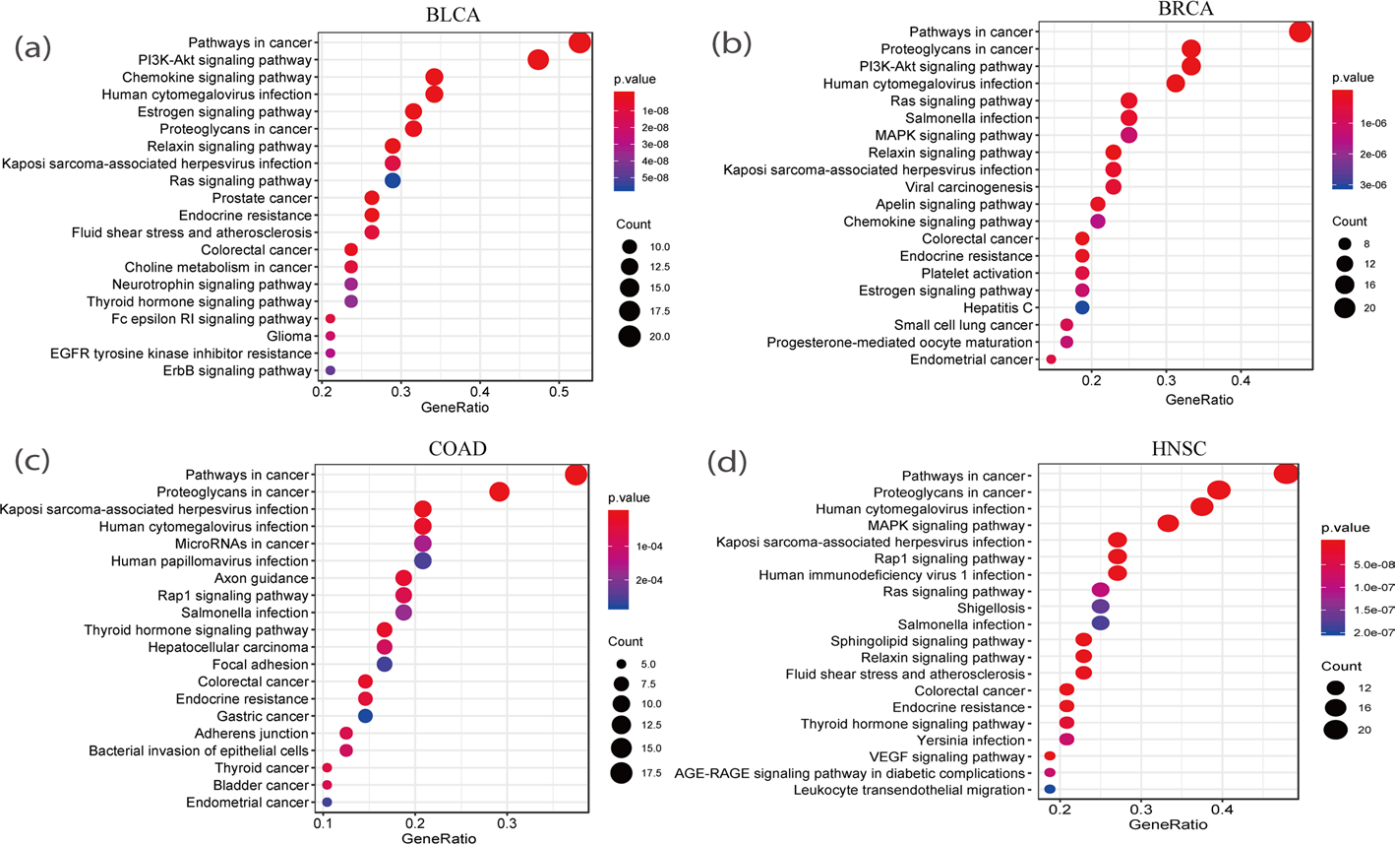
Results of KEGG pathway enrichment analysis on top 50 ranking candidate driver genes predicted with our PDGPCS for BLCA, BRCA, COAD and HNSC cancers. **a **BLCA, **b **BRCA, **c **COAD, and **d **HNSC

To test whether the predicted driver genes of PDGPCS are related with specific biological functions or pathways, we performed KEGG pathway enrichment analysis on top 50 ranking candidate driver genes predicted with PDGPCS by using R package clusterProfiler [22]. The results of pathway enrichment analysis on four cancer datasets were shown in Fig. 14. As shown in Fig. 14, we can see that most significant pathway among top 20 ranking pathways for BLCA, BRCA, COAD and HNSC is “pathways in cancer”, demonstrating that the predicted driver genes with our PDGPCS are significantly related with cancers. We also identified other cancer-related pathways for BLCA, BRCA, COAD and HNSC, such as “PI3K-Akt signaling pathway” [23] and “ErbB signaling pathway” [24] for BLCA, “MAPK signaling pathway” [25] and “Jak-STAT signaling pathway” [26] for BRCA, “Rap1 signaling pathway” [27] for COAD, and “VEGF signaling pathway” for HNSC [28].

### Survival analysis for PDGPCS

To further provide more biological evidences, we performed survival analysis on top 50 ranking candidate driver genes predicted with PDGPCS for four cancers by using an online tool GEPIA2 [29]. GEPIA2 (http://gepia2.cancer-pku.cn/#survival) can support the survival analysis on TCGA cancer datasets by providing gene symbols in multiple cancer types, and led to the identification of potential biomarkers. Furthermore, we also analyzed the survival results on top 50 ranking candidate driver genes predicted with IMCDriver on four cancers by using an online tool GEPIA2 [30]. The survival results of PDGPCS and IMCDriver on four cancers were shown in Additional file 6, from which we can see that for BLCA, BRCA, COAD and HNSC cancers, our PDGPCS identified 7, 9, 4 and 7 significant biomarker genes with logrank *P*-value < 0.05, while IMCDriver identified 5, 2, 4 and 8 significant biomarker genes. Furthermore, among these significant survival genes, PDGPCS identified 5, 8, 3 and 5 novel biomarker genes, while IMCDriver identified 1, 2, 2 and 1 novel significant biomarker genes (i.e., significant biomarker genes not in CGC dataset). Therefore, for cancer survival analysis, PDGPCS performs better than IMCDriver on BLCA, BRCA, COAD and HNSC cancer data sets. It is worth noting that IMCDriver is a supervised method that depends on the previous known driver genes for identifying personalized driver genes, and may ignore other important driver genes not within the previous known driver genes (e.g., some meaningful biomarker genes not within the previous known driver genes for survival analysis). Therefore, PDGPCS as an unsupervised method can discover complementary meaningful biomarker genes for survival analysis compared with IMCDriver method. In addition, Additional file 1: Figs. S2–S5 gave the survival analysis curves of the biomarker genes for BLCA, BRCA, COAD and HNSC, respectively. These results showed that our PDGPCS can effectively predict the cancer biomarker genes.

## Conclusions

Considering the edge weight information of gene interactions for individual cancer patients, we proposed a novel method (namely PDGPCS) to predict the cancer driver genes by building the prize-collecting Steiner tree on personalized weight gene interaction network. PDGPCS can predict the personalized driver genes of individual patients. Since the gold-standard of the personalized driver genes is not available, here we used Condorcet voting method on PDGPCS for obtaining the prediction results of PDGPCS in the entire cohorts. Thus we evaluated our PDGPCS’s performance with the gold-standard of cancer driver genes for the entire cohorts (i.e., Cancer Genes census). Firstly, we used the gene expression data from the cancer tissues and normal tissues of individual cancer patients to construct a personalized weighted gene interaction network, taking advantage of the individual gene expression data to measure the weights of edges. Then, we identified the differentially expressed genes and dysregulated pathways in the personalized weighted gene interaction network, screening the reliable mutant genes. Thirdly, the mutant genes, dysregulated pathways and individual differential co-expression networks were used to construct a mutation-dysregulation network, and the prize-collecting Steiner tree model was adopted to quantify the influence of mutant genes on dysregulated pathways. In the end, PDGPCS realized the cancer drive gene prediction. The experimental results on four cancers show that: (1) PDGPCS has a better prediction performance than other existing individual driver gene prediction methods; (2) it is effective by using the strategy of using the individual gene expression data to assign edge weights; (3) when cancer patients have the paired normal and tumor sample data, using the paired samples approach can improve the performance of DGPCS; (4) the cancer driver genes predicted with PDGPCS are significantly enriched in the pathways related with cancers, and some driver genes can significantly affect the survival of cancer patients.

In conclusion, compared with other existing methods, the main advantage of our PDGPCT in handling the patient-specific network is that it focuses on both patient-specific node and edge weight information. In contrast, most of existing methods mainly consider the patient-specific nodes information and ignore the patient-specific edges (i.e., gene interactions) information. The experimental results also validated that the edge weight in the personalized gene interaction network can improve the performance of PDGPCS. However, our PDGPCS predicts the cancer driver genes by constructing an undirected network, and ignores the directed transmission information of edges in biological networks. Therefore, extending the PCST model to a directed network and studying the regulation mechanism of cancer driver genes on pathways is one of the directions for future improvement.

## Materials and methods

### Datasets

The datasets mainly consist of the personalized multi-omics data of individual patients, gene/protein interaction data and pathway data. The muti-omics data of individual patients are two types of genomic data (i.e., gene expression and gene mutation) of 189 cancer patients on 4 cancers from the TCGA data portal. The gene expression data includes the cancer sample data and normal sample data for each individual patient, and the gene mutation data contains the single nucleotide variation (SNV) data and copy number variation (CNV) data. For each patient, we collected the gene expression data of paired tumor and normal samples and gene mutation data (i.e., SNV and CNV) of tumor samples. We used gene mutation data (i.e., SNV and CNV) of individual samples which were collected by Bertrand et al [30]. The CNV_data contains a tab-separated file with individual patients as columns and genes as rows. Each entry of this matrix holds either a value of − 1/0/1. A value of − 1/1 indicates a deletion/amplification event in the gene of that particular individual’s sample. The SNV_data contains of a tab-separated file with individual patients as columns and genes as rows. Each entry of this matrix holds either the binary values of 0/1. The value 1 indicates the presence of a single nucleotide variation in the gene of that particular individual’s sample. For SNV data and CNV data of each column (i.e., each individual patient), we considered the gene with nonzero value as the mutated genes.

These 189 cancer patients from 4 cancers consist of 19 patients for bladder cancer (BLCA), 100 patients for breast cancer (BRCA), 27 patients for colon cancer (COAD), 43 patients for head and neck squamous cell carcinoma (HNSC). Gene/protein interaction network data was taken from STRING v10.5 [31]. We used only the physical interactions that were validated experimentally, and interactions from other curated databases with confidence score > 0.7 [1]. The network consists of 11,289 genes and 273,210 edges. The pathway data was collected from curated Kyoto Encyclopedia of Genes and Genomes (KEGG) dataset, and the Pathview tool [32] was used to extract 244 pathways in total.

### PDGPCS algorithm

The biological motivation of PDGPCS consists of two respects as follows: (1) the regulation weight of personalized driver genes varies among individual patients during the phase transition between normal state and tumor state; (2) the influence of driver genes is disseminated along pathways and is manifested by differentially expressed genes on the personalized weighted gene interaction network [1]. Figure 1 is the schematic diagram of PDGPCS, which is described in detail as follows.

### Step 1 Construct the personalized gene interaction network

For an individual patient, we used paired-SSN method [3] for constructing the personalized gene interaction network. The Paired-SSN uses Single Sample Network (SSN) method [33] to obtain the co-expression *P*-values of the gene interactions for normal sample and tumor sample, respectively. The *P*-value of an edge for a single sample can be obtained from the statistical Z-score by measuring Δ*PCC*. Δ*PCC* and *Z*-score of an edge between genes $i$ and $j$ are calculated with the following formulas:

1 $$\Delta PCC_{ij} = PCC_{ij}^{n + 1} - PCC_{ij}^{n}$$

2 $$Z_{ij} = \frac{{\Delta PCC_{ij} }}{{{{\left( {1 - \left( {PCC_{ij}^{n} } \right)^{2} } \right)} \mathord{\left/ {\vphantom {{\left( {1 - \left( {PCC_{ij}^{n} } \right)^{2} } \right)} {\left( {n - 1} \right)}}} \right. \kern-\nulldelimiterspace} {\left( {n - 1} \right)}}}}$$

where $PCC_{ij}^{n}$ is the Pearson Correlation Coefficient (PCC) between gene $i$ and $j$ in the gene expression profile matrix of *n* reference samples, and $PCC_{ij}^{n + 1}$ is the PCC between gene $i$ and $j$ in the perturbed gene expression profile matrix which contains *n* reference samples (i.e., all normal samples) and one additional sample (i.e., normal sample or tumor sample for each patient). Based on the above equation, we can use Z-test [34] to calculate the *P*-value of each edge in the gene interaction network.

To illustrate how much by adding just one gene expression to the existing gene expression data sets will affect the amount of Pearson correlation coefficient (PCC) between genes, we simulated the distribution of PCC perturbation (*i.e*., Δ*PCCn*) when adding just one gene expression to the existing gene expression data sets. The PCC perturbation Δ*PCCn* can be calculated with the Eq. 1. Firstly, we generated two series of reference numbers, and the correlation of the two series of numbers was a fixed value (*PCCn* = 0). The length *n* of the two series (*i.e*., the number of the reference samples) was chose as 20, 50, 100 and 200. Based on the generated two series of reference numbers, we randomly generated one series of two numbers (gene expression value of two genes for one sample) and obtained the distribution of PCC perturbation Δ*PCCn.* The Δ*PCCn* demonstrates the perturbed degree value when adding just one gene expression to the existing gene expression data sets affects the amount of Pearson correlation between genes. The random simulation was repeated 2,000,000 times, where the value of Δ*PCCn* with a significant *P*-value of 0.05 in the two-tails area was selected from every distribution of simulation (Additional file 1: Fig. S6). As is shown in Additional file 1: Fig. S6, we can see that: (1) the range of Δ*PCCn* decreases when the number of reference samples increases; (2) the distribution of Δ*PCCn* can be approximated by a normal distribution. Based on simulated distribution of Δ*PCCn* (Eq. 1) (Additional file 1: Fig. S6), the Z-score of Δ*PCCn* with a significant P-value of 0.05 in the two-tails area could be obtained.

To further obtain the simulational Z-score of Δ*PCCn* for different values of PCCn, we firstly divided PCCn = [− 1:1] into ten intervals uniformly. Then we randomly selected a value from each interval to simulate the simulated distribution of Δ*PCCn*. Based on simulated distribution of Δ*PCCn*, the Z-score of Δ*PCCn* with a significant P-value of 0.05 in the two-tails area could be obtained for a value of each interval. We repeated this process 100 times and obtained the average simulational values for different intervals and theoretical results for Z-score of Δ*PCCn* with a significant *P*-value of 0.05 in the two-tails area (Additional file 1: Fig. S7). In fact, we can obtain the theoretical value for Z-score of Δ*PCCn* with a significant *P*-value of 0.05 in the two-tails area with the Eq. 2.

As shown in Additional file 1: Fig. S7, the Z-scores of the simulation and the theoretical calculation have little difference when PCCn is larger than − 0.04. However, when PCCn is less than − 0.04, there is a big difference between the simulation and the theoretical calculations. By deleting the edges with PCCn < -0.4 in the personalized gene interaction network (PGIN), we reran our PDPCS on BLCA, BRCA, COAD and HNSC cancer data sets and obtained the corresponding results of precision, recall and F-score among top *k* (*k* = 1,2,3,…,50) ranking driver genes (Additional file 1: Figs. S8–S10). Additional file 1: Figs. S8–S10 show that deleting the edges with PCCn < − 0.4 will improve the prediction results for COAD and HNSC cancer data sets. Therefore, in the future, we can develop effective strategies for SSN method to delete these noise of PGIN.

We should note that the Z-score of Eq. 2 is no longer valid if the Pearson correlation between gene *i* and gene *j* in the gene reference state is + 1 or − 1. Therefore, in this study, our PDGPCS removed these edges whose Pearson correlations in the gene reference state are + 1 or − 1. To demonstrate the effect of this procedure on the discovery of cancer driver genes, we extracted the sub-network whose Pearson correlation in the gene reference state is + 1 or − 1, and then calculated the ratio of these nodes to all nodes in the gene interaction network on four cancer datasets (Additional file 1: Fig. S11). As shown in Additional file 1: Fig. S11, we can see that the number of genes related with edges whose Pearson correlation in the gene reference state is + 1 or − 1 only account for minority on four cancer datasets. For example, for BLCA and HNSC, the number of genes account for less than 1% and there are no genes whose Pearson correlation in the gene reference state is + 1 or − 1 for BLCA and HNSC.

For BLCA and HNSC, we also calculated the P-value of these genes enriched in cancer census genes by hyper-geometric test,

3 $$p{ - }value = \sum\limits_{{o_{i} = o_{s} }}^{o} {p(o,o_{i} )}$$

4 $$p(o,o_{i} ){ = }\frac{{\left( \begin{gathered} s \hfill \\ o_{i} \hfill \\ \end{gathered} \right)\left( \begin{gathered} N - s \hfill \\ o - o_{i} \hfill \\ \end{gathered} \right)}}{{\left( \begin{gathered} N \hfill \\ o \hfill \\ \end{gathered} \right)}}$$

where *N* denotes the total number of genes in the gene interaction network; *o* is the number of genes related with edges whose Pearson correlation in the gene reference state is + 1 or − 1; *s* is the number of intersected genes between these genes with *PCCn* = 1 or − 1 and the gold standard driver genes from the Cancer Gene Census (CGC). The *P*-value results of these genes enriched in cancer census genes were shown in Additional file 1: Fig. S12. Additional file 1: Fig. S12 shows that *P*-values of these genes enriched in cancer census genes on BLCA and HNSC are larger than 0.05, indicating that removing these edges whose Pearson correlations in the gene reference state are + 1 or − 1 does not affect the discovery of cancer driver genes.

According to co-expression *P*-value of the gene interactions from normal sample and tumor sample, we constructed the personalized gene interaction network for each patient by using the paired-SSN method [3] and the known PPI network [1]. In the personalized gene interaction network, if the co-expression *P*-value of the interaction edge in gene interaction network of tumor sample is less than (or greater than) 0.05, and that greater than (or less than) 0.05 in the gene interaction network of normal sample, there is an edge between gene $i$ and gene $j$. That is, we calculated the co-expression differences *P*-value of the edges in known PPI network between normal and tumor samples for an individual patient, and then extracted the overlap between significant co-expression edges and known gene/protein interaction network. Therefore, a personalized gene interaction network can reflect the gene interactions with significant differences between normal and tumor samples of an individual patient.

Taking the individual differences into account, we calculated the edge weight $w_{ij}$ between gene *i* and *j* to construct the personalized weighted gene interaction network $G = \left( {V,E} \right)$.

5 $$w_{ij} = \left| {\log_{2} \left| {\frac{{\left( {\Delta PCC_{ij} } \right)_{Tumor} }}{{\left( {\Delta PCC_{ij} } \right)_{Normal} }}} \right|} \right|$$

**Fig. 15:**
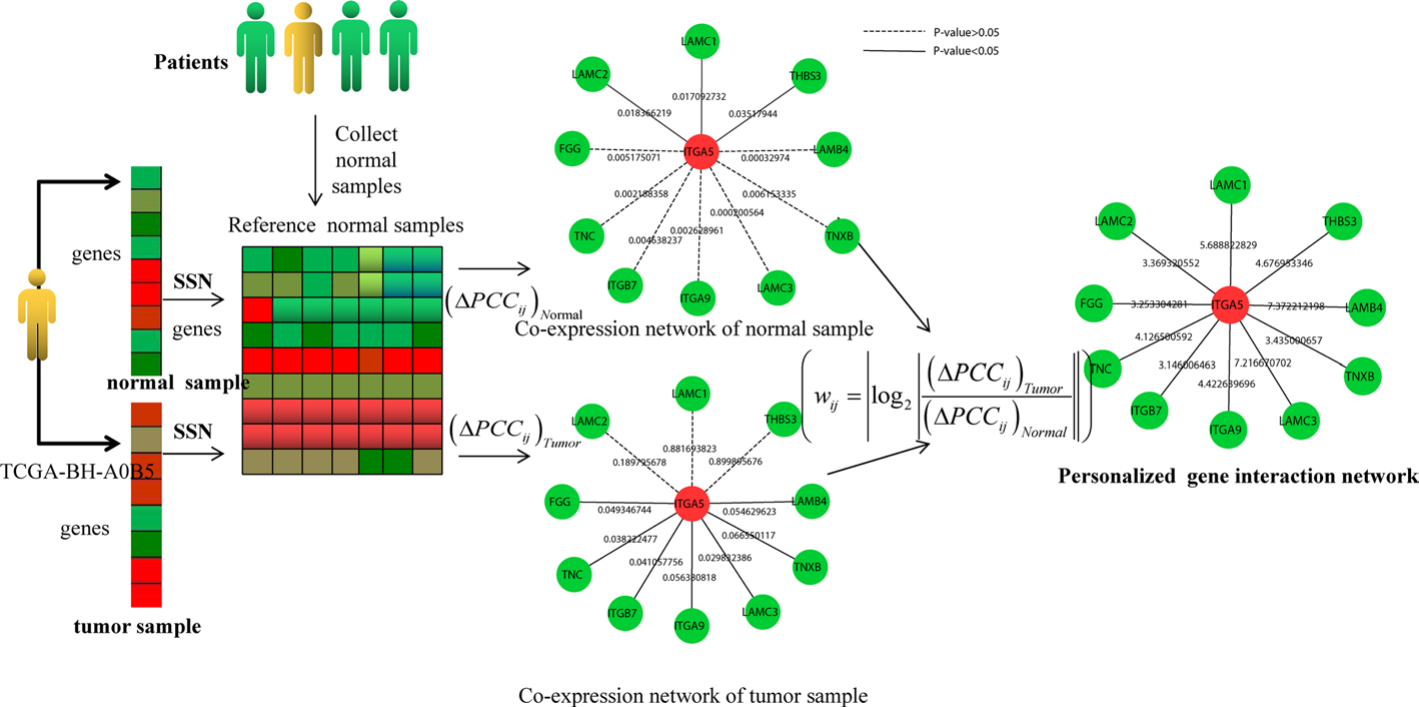
Overview of paired-SSN for constructing personalized weight gene interaction networks for a given cancer patient, TCGA-BH-A0B5 in BRCA cancer data. For a given cancer patient, we firstly chose the expression data of all normal samples in BRCA as the reference data and constructed the co-expression network of tumor sample (white color) and normal sample (green color) respectively with the reference data by using SSN method. Then the personalized weighted gene interaction network was constructed by using significant interactions where the co-expression *P*-value of of tumor sample is less than (or greater than) 0.05, and that greater than (or less than) 0.05 of normal sample. Furthermore, we extracted the edge weights of significant interactions by doing the log_2_ operation on the ratio of the two weights. The edge weights indicate transition degree value of significant personalized gene interactions between the normal state and tumor state of an individual patient. Here we showed the individual specific sub-networks related with driver gene ITGA5 which contain its first-order neighboring genes as an example

Furthermore, in Fig. 15, we give a example how to construct the personalized weighted gene interaction network based on the expression data of a cancer patient, TCGA-BH-A0B5 in BRCA cancer data.

### Step 2 Identify the dysregulated pathways and the key mutant genes of individual patients

Given the gene expression data (including tumor and normal) of an individual patient, differentially expressed genes (DEGs) are identified by calculating the fold change between normal sample and tumor sample. That is, all gene with $\left| {\log_{2} fold \, change} \right| > \beta$(here, we set $\beta = 1$) are identified as DEGs, and the DEGs set is represented as $DEG$. The hypergeometric distribution test is used to conduct pathway enrichment analysis for DEGs. The pathways with significantly enriched DEGs (*FDR* < 0.05) are considered as the potential dysregulation pathways, and the set of all the potential dysregulation pathways is represented as $DP$.

To screen the key mutant genes in the personalized gene interaction network, we extracted all mutated genes from gene mutation data (i.e., SNV and CNV) of tumor samples, and selected the personalized significant co-mutated genes which tend to promote tumorigenesis and anti-disease drug responses [35] as key mutant genes of an individual patient. In detail, we firstly took all the mutant genes of an individual patient as the initial seeds, and implemented random walk with restart (RWR) algorithm on the personalized gene interaction network to calculate the personalized co-mutation score (or co-mutation probability) of each mutated gene reached from the individual mutations. Then, we generated 50 topologically matched random networks, each of which maintains the topological characteristics (e.g., degree distribution) in the original personalized gene interaction network. RWR was also implemented on the 50 random networks to null distribution of co-mutation score of each mutant gene reached from the individual mutations, and the randomization-based test [36] was used to evaluate the statistical significance for the potential mutant genes (see the details in Supplemental Methods of Supplemental File A). Finally, the candidate mutant genes with statistical significance (i.e.,$p{ - }value < 0.05$) are considered as the candidate personalized co-mutant genes, and the candidate co-mutant genes set is represented as *K*. Considering the case that hub genes in the personalized gene interaction network can be regarded as the potential driver genes, we screen the hub genes by fixing a threshold ($\alpha$) of the node degree (i.e., the mean value of node degree) in the personalized gene interaction network. The set of the screened hub genes is represented as *H.* We regard the intersection of *K* and *H* as the key mutant genes *M*, i.e., $M = K \cap H$.

In order to illustrate that using 50 random networks as the null model is feasible, we randomly generated 100 random networks, then implemented the random walk with restart (RWR) algorithm on these random networks to obtain the walk probability of each mutant gene in these 100 random networks. For each mutant gene, we calculated its *P*-*value* that demonstrates whether the walk probability distribution of 50 random networks is different from that of 100 random networks by using Kolmogorov–Smirnov test [37]. If the *P*-value of a mutated gene is larger than 0.05, we consider that the walk probability distribution of 50 random networks and 100 random networks follow the same distribution. Thus, we calculated the proportion of mutant genes with *P*-value > 0.05 between 50 random networks and 100 random networks. Similarly, we also randomly generated 150 and 200 random networks to calculate their corresponding proportion of mutant genes with *P*-value > 0.05 when taking 50 random networks as a reference. The results were shown in (Additional file 1: Fig. S13). Taking BRCA as an example (in Additional file 1: Fig. S13), there are more than 97% mutant genes following the same distribution between 50 random networks and 100, 150, 200 random networks. These results demonstrated that there did not exist significant difference for most nodes between 50, 100, 150 and 200 random networks, and it is reasonable for considering 50 random networks as null models for screening the key mutant genes.

### Step 3 Prioritize the personalized cancer driver genes

To prioritize the personalized driver genes, PDGPCS uses the Prize-Collecting Steiner Tree (PCST) model to find a subnetwork (or tree) $$T = \left( {V_{t} ,E_{t} } \right)$$ with root mutation gene $${\text{g}}_{root}$$$$({\text{g}}_{root} \in V_{t} )$$ which offers an interpretation of how the mutant gene causes its downstream gene dysregulation on a given personalized weighted network [38, 39]. That is, if the root mutation gene $${\text{g}}_{root}$$ is a driver gene, then it will cause a lot of gene dysregulation in subnetwork *T* with maximizing the sum of node prize and minimizing the sum of edge cost [1]. In PDGPCS, the edge cost reflects the differential extent of two gene co-expression in the condition of tumor and normal samples. The greater the difference, the smaller the cost. And the node prize is only awarded to differentially expressed genes, whereas other non-differentially expressed genes that serve as the intermediate nodes (i.e., Steiner nodes) in the subnetwork *T* are not assigned with the weights.

In detail, we firstly used the dysregulated pathway $p = \left( {V_{p} ,E_{p} } \right)$($p \in DP$) and the key mutant gene $g$($g \in M$) to obtain the weighted mutation-dysregulation network $G_{p,g} = \left( {V_{p,g} ,E_{p,g} } \right)$ on the personalized weighted gene interaction network $G = \left( {V,E} \right)$. We should note that we consider the personalized weighted network as undirected and weighted network $G = \left( {V,E} \right)$.Here, $V_{p,g} = V_{p} \cup g \cup N_{h}$, $N_{h}$ represents the set of the nearest neighbor genes of $V_{p}$ genes and key mutant gene $g$; $E_{p,g} = E_{p} \cup \left\{ {\left. {\left( {u,v} \right)} \right|u \in V_{p,g} ,v \in V_{p,g} ,\left( {u,v} \right) \in E} \right\}$, i.e., the union set of edges of personalized gene interaction network whose nodes are within the mutation-dysregulation network and edges in the dysregulated pathway. For network $G_{p,g}$, we only assigned the weight values for DEGs. Therefore, the prize for node *v* in $G_{p,g}$ is defined as follow:

6 $$P_{p,g} \left( v \right) = \left\{ {\begin{array}{*{20}l} {\left| {\log_{2} fold \, change} \right|,v \in DEG \cap V_{p} } \hfill \\ {0,{\text{ otherwise}}} \hfill \\ \end{array} } \right.$$

According to the criterion that the greater the edge weight, the smaller the edge cost, the cost $c(e_{ij} )$ of edge $e_{ij}$($e_{ij} \in E_{p,g}$) is defined as follow:

7 $$c(e_{ij} ) = \left\{ {\begin{array}{*{20}l} {1 - norm(w_{ij} ), \, e_{ij} \in E \cap E_{p,g} } \hfill \\ {1 - \max (norm(W)),e_{ij} \in E_{p} ,e_{ij} \notin E} \hfill \\ \end{array} } \right.$$

where $w_{ij}$ is the weight of edge $e_{ij}$, $W$ is the weights of all edges_;_ $norm(w_{ij} )$ represents normalization of $w_{ij}$, i.e., $norm(w_{ij} ) = (w_{ij} - \min (W))/(\max (W) - \min (W))$ and $norm(W)$ represents the normalization of all edges in *W*. $E$,$E_{p}$,$E_{p,g}$ denote the set of edges in the personalized gene interaction network, dysregulated pathway and weighted mutation-dysregulation network, respectively. To prefer the original pathway edges, we set the cost of these edges in pathway but not in personalized weighted gene interaction network as a small value.

Then, we adopted PCST model on network $G_{p,g}$ to search the dysregulation subnetwork *T* by setting the key mutant gene *g* as a root node. The subnetwork *T* satisfies the following optimization functions:

8 $$\max f(T) = \sum\limits_{{v \in V_{t} }} {p\left( v \right)} - \sum\limits_{{e \in E_{t} }} {c\left( e \right)}$$

where $\max f(T)$ refers to maximizing the sum of node prize minus the sum of edge cost in the subnetwork *T*, $p\left( v \right) \in R^{ + }$ represents the prize of node *v*, and $c\left( e \right) \in R^{ + }$ represents edge cost in $G = \left( {V,E} \right)$. If the solution of PCST exists, its corresponding subnetwork *T* can be interpreted as the influence of the mutant gene to the its downstream differentially expressed genes (DEGs) on the dysregulated pathway *p*. The influence score of mutant gene *g* to its downstream DEGs on dysregulated pathway *p* is defined as $infl\left( {p,g} \right) = {{f_{g} (T)} \mathord{\left/ {\vphantom {{f_{g} (T)} {\sum\limits_{{v \in DEG \cap V_{p} }} {P_{p,g} \left( v \right)} }}} \right. \kern-\nulldelimiterspace} {\sum\limits_{{v \in DEG \cap V_{p} }} {P_{p,g} \left( v \right)} }}$, where $f_{g} (T)$ is the PCST solution, $P_{p,g} \left( v \right)$ denotes the prize for node *v* ($v \in DEG \cap V_{p}$) in the weighted mutation-dysregulation network $G_{p,g}$. In fact, PCST can generate an optimized subnetwork when root node is not an isolated node in the network. The weights of the node and the costs of the edge on network *G* was shown in Additional file 1: Fig. S14. The blue nodes are called Steiner node, and they have no weights. If we took node ‘*a*’ (red) as the root node, we can obtain six different subnetworks and calculate the values (i.e., 0.7, 1.9, 2.1, 1.8, 3.3, 3) of these subnetworks, respectively. Therefore, the optimized subnetwork is the result of (E) and the corresponding weight is *f*(*T*) = 3.3. However, when node ‘*a*’ is an isolated node (Additional file 1: Fig. S15), we implemented the PCST algorithm with ‘*a*’ as the root node in this network, the subnetwork cannot be generated.

Finally, by repeating above operation for each pathway and each mutant gene, we can obtain the influence score matrix of the mutant genes on the dysregulated pathways. The sum of influence scores of mutant gene *g* on all dysregulated pathways is defined as the total influence score $infl\left( g \right)$ of mutant gene *g*, that is,

9 $$infl\left( g \right) = \sum\limits_{p \in DP} {infl\left( {p,g} \right)}$$

According to the total influence scores of all mutant genes, we ranked the mutant genes in descending order, and then selected the top ranked mutant genes as the driver genes of individual cancer patient.

### Assessment metrics

Considering that the gold-standard of the personalized driver genes is not available, we selected top 50 ranked mutant genes in the population as the candidate driver genes to assess the performance of different prediction methods. Here we used a curated list of driver genes from the Cancer Gene Census (CGC) as the gold standard benchmark data [16]. It includes a list of 616 cancer genes. The full list of genes in CGC were provided in Additional file 2. The precision, recall and F1 were used to measure the performance of prediction methods. The equations for calculating precision, recall and F1 are listed as follows,

10 $$precision(k) = \frac{{\left| {rank(k) \cap CGC} \right|}}{k}$$

11 $${\text{Re}} {\text{call}}(k) = \frac{{\left| {rank(k) \cap CGC} \right|}}{{\left| {Muated \cap CGC} \right|}}$$

12 $$F1(k) = 2/(1/precision(k) + 1/{\text{Re}} call(k))$$

where *rank*(*k*) denotes the ranking of driver genes for a given method; *CGC* denotes the gold standard driver genes from the Cancer Gene Census (CGC); *Mutated* denotes the mutated genes from Single nucleotide variation (SNV) data and copy number variation (CNV) data.

## Supplementary Information

**Additional** **file 1.** Supplemental method and figures.

**Additional** **file 2.** Results of ranking genes for PDGPCS, PRODIGY and three centrality measures methods on BLCA, BRCA, COAD and HNSC cancers, respectively. In addition, we provided the full list of the gold standard driver genes from the CGC dataset. 

**Additional** **file 3.** The exact values of the precision, recall and F1-score of our PDGPCS, PRODIGY and three centrality measures methods on BLCA, BRCA, COAD and HNSC cancers, respectively.

**Additional** **file 4.** The exact values of the precision, recall and F1-score of our assigned edge weight strategy of PDGPCS and random weight strategy on BLCA, BRCA, COAD and HNSC cancers, respectively.

**Additional** **file 5.** The exact values of the precision, recall and F1-score of Paired-SSN and SSN on our PDGPCS on BLCA, BRCA, COAD and HNSC cancers, respectively.

**Additional** **file 6.** Survival analysis results for top50 ranking driver genes of PDGPCS and IMCDriver on four cancers by using an online tool GEPIA2.

## Data Availability

The datasets generated and/or analysed during the current study are available in the Github repository, https://github.com/NWPU-903PR/PDGPCS.

## Abbreviations

SSN: Single sample network
RWR: Random walk with restart
PCST: Prize-collecting Steiner tree
BLCA: Bladder urothelial carcinoma
COAD: Colon adenocarcinoma
HNSC: Head and neck squamous cell carcinoma
BRCA: Breast invasive carcinoma
SNV: Single nucleotide variation
CNV: Copy number variation
CGC: Cancer gene census
KEG: Kyoto encyclopedia of genes and genomes
PCC: Pearson correlation coefficient
DEG: Differential expression gene
DP: Dysregulation pathway
