# Supplementary material for: Prioritization of cancer driver gene with prize-collecting steiner tree by introducing an edge weighted strategy in the personalized gene interaction network
Source: BMC Bioinformatics. 2022 Aug 16;23:341. doi: 10.1186/s12859-022-04802-y (PMC9380343; doi:10.1186/s12859-022-04802-y)
Supplement: Supplementary file 1 — Additional file 1. Supplemental method and figures. [file 12859_2022_4802_MOESM1_ESM.docx]

Supplemental Method and Figures

**Prioritization of cancer driver gene on the personalized weighted gene interaction network with Prize-Collecting Steiner Tree**

Shao-Wu Zhang^1^*, Zhen-Nan Wang^1^, Yan Li^1^, Wei-Feng Guo^2^ *

^1^ Key Laboratory of Information Fusion Technology of Ministry of Education, School of Automation, Northwestern Polytechnical University, Xian 710072, China

^2^ School of Electrical Engineering, Zhengzhou University, Zhengzhou 450001, China

^*^ Corresponding author(s). Email: [zhangsw@nwpu.edu.cn](mailto:zhangsw@nwpu.edu.cn); [guowf@zzu.edu.cn](mailto:zengtao@sibs.ac.cn,%20lnchen@sibs.ac.cn)

Supplementary method

## Random walk with restart algorithm to search individual mutant genes

RWR (random walk and restart) algorithm simulates a random walker’s transition in the network from a starting node (or a few starting nodes), with the predefined starting probabilities, to its neighbors until it reaches a stable state. RWR allows for revisiting the starting node(s) with revisiting probabilities. The RWR algorithm is formulated as follows,

$$p^{t+1}=(1-r)Wp^{t}+rp^{t}$$

where *r* is the restart probability (here we set *r*=0.6 in this work); W is denoted as the column-normalized adjacency matrix for a network *G* with *n* nodes; *p*^0^, *p^t^* or *p^t^*^+1^, represents a vector in which the *i*-th element holds the probability that the walker is at node *i* at time steps 0, *t* or *t*+1 respectively. In general, assuming that there are *k* initial genes from which the walker would start with equal probability, the initial vector *p*^0^ is defined as a vector, with initial nodes having a probability of 1/*k* and the remaining nodes having a probability 0. The RWR function is solved using this iteration process when the difference between *p^t^* and *p*^t+1^ is below a predefined threshold (e.g., 10^-6^ in our analysis).

To confirm how many nodes (genes) of the sub-network are generated by RWR, we generate 100 random networks, each of which have the topological characteristics (i.e., degree of each node) of the original network, and recalculate the walk probability of each node from the initial nodes. The random data sets were used to obtain an empirical null distribution for the walk probability for each node. Then we compute z-score as

$$z^{i}=\frac{p_{i}-mean(SD_{i})}{std(SD_{i})}$$

where *p_i_* is the walk probability for the node *i* from the initial nodes in the network. *SD_i_* is the distribution of the walk probability of node *i* generated in the random networks. Mean and *std* of *SD_i_* are computed from 100 simulations of random networks.

Note that the entire gene network with 11289 genes and 273210 edges is used as the background network for random sampling. Based on z-score, we can obtain the empirical *p*-value *p_i_* (modeled as Gaussian distribution). In each patient, we iteratively took each mutation gene as the starting point to initiate the random walk and the genes with *p_i_*<0.05 are retained and denoted as significant genes for the mutation genes. The mutation genes and the significant genes, together with their interactors are formed into the individual mutated network.

## Condorcet voting for gene rank aggregation in population

To aggregate the ranking of genes from individual patient samples for determining the most impactful drivers in a population, PDGPCS applied the Condorcet method ^[1](file:///C:\\Users\\42114\\Desktop\\bty006-suppl_data_SCS\\Supplementary%20manuscript.docx#_ENREF_1)^. The Condorcet method is a voting scheme in which ‘voters’ (patients) vote for the best ‘candidate’ by submitting a rank-ordered list of candidate preferences (i.e., ranked driver mutations). The list of preferences is allowed to be either partial or full. The Condorcet method then selects a winning candidate by comparing every possible pair of candidate G_A_ and G_B_ for each patient and determines a ‘winner’ by comparing the number of voters that preferred G_A_ and G_B_ and vice versa in the population. Since PDGPCS can output a ranking as an impact score for the candidate driver mutation genes for the patients, we applied the Condorcet method to evaluate pairwise comparisons of two candidate driver mutation genes in the population.

Supplementary Figures

**Figure S1** Precisions, Recalls and F1-scores of PDGPCS, PRODIGY, SCS and three centrality measures methods (Degree, Betweenness and Closeness) for predicting the driver genes using Reactome pathways on (a) Precision, Recall and F1-score for BLCA cancer, (b) Precision, Recall and F1-score for BRCA cancer, (c) Precision, Recall and F1-score for COAD cancer, (d) Precision, Recall and F1-score for HNSC cancer.

**Fig. S2** Survival analysis curves of 7 biomarker genes predicted with our PDGPCS for BLCA cancer. (a) RAC3 gene, (b) MAPK1 gene, (c) PRKAR2A gene, (d) CAD gene, (e) SRC gene, (f) P4HB gene, (g) ITGB8 gene.

**Fig. S3** Survival analysis curves of 9 biomarker genes predicted by our PDGPCS for BRCA cancer. (a) SHMT2. (b) TXN. (c) NOS2. (d) TRAF2. (e) PYCARD. (f) SDC1. (g) COPS5. (h) SOD1. (i) TP53.

**Fig. S4** Survival analysis curves of 4 biomarker genes predicted by our PDGPCS for COAD cancer. (a) TKT. (b) CDC42. (c) NOTCH3. (d) TP53

**Fig. S5** Survival analysis curves of 7 biomarker genes predicted by our PDGPCS for HNSC cancer. (a) EPRS. (b) NT5E. (c) FADD. (d) LAMB3. (e) HSP90AA1. (f) CYCS. (g) MYC.


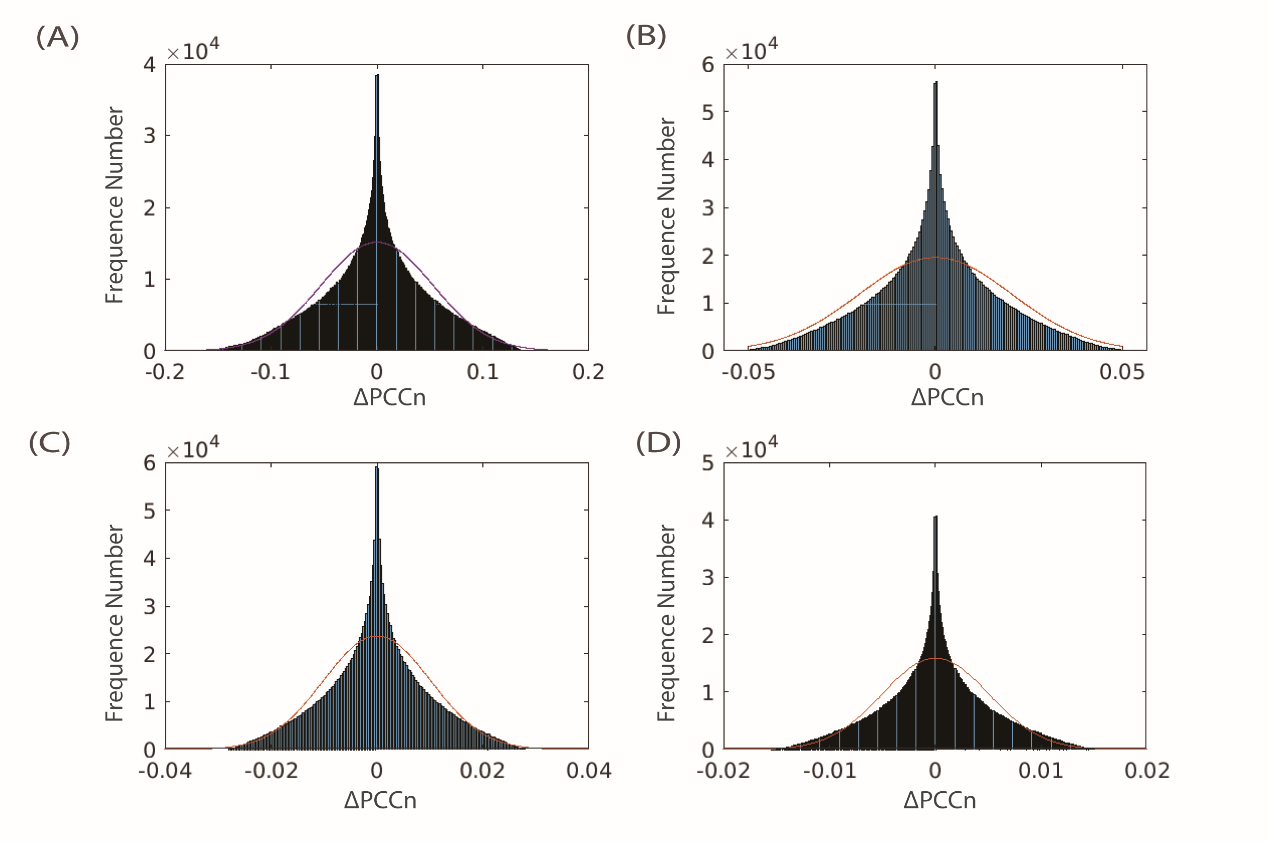


Fig. S6 The distribution of ΔPCCn numerically obtained by random simulation for (A-D) n=20, 50 , 100 and 200.


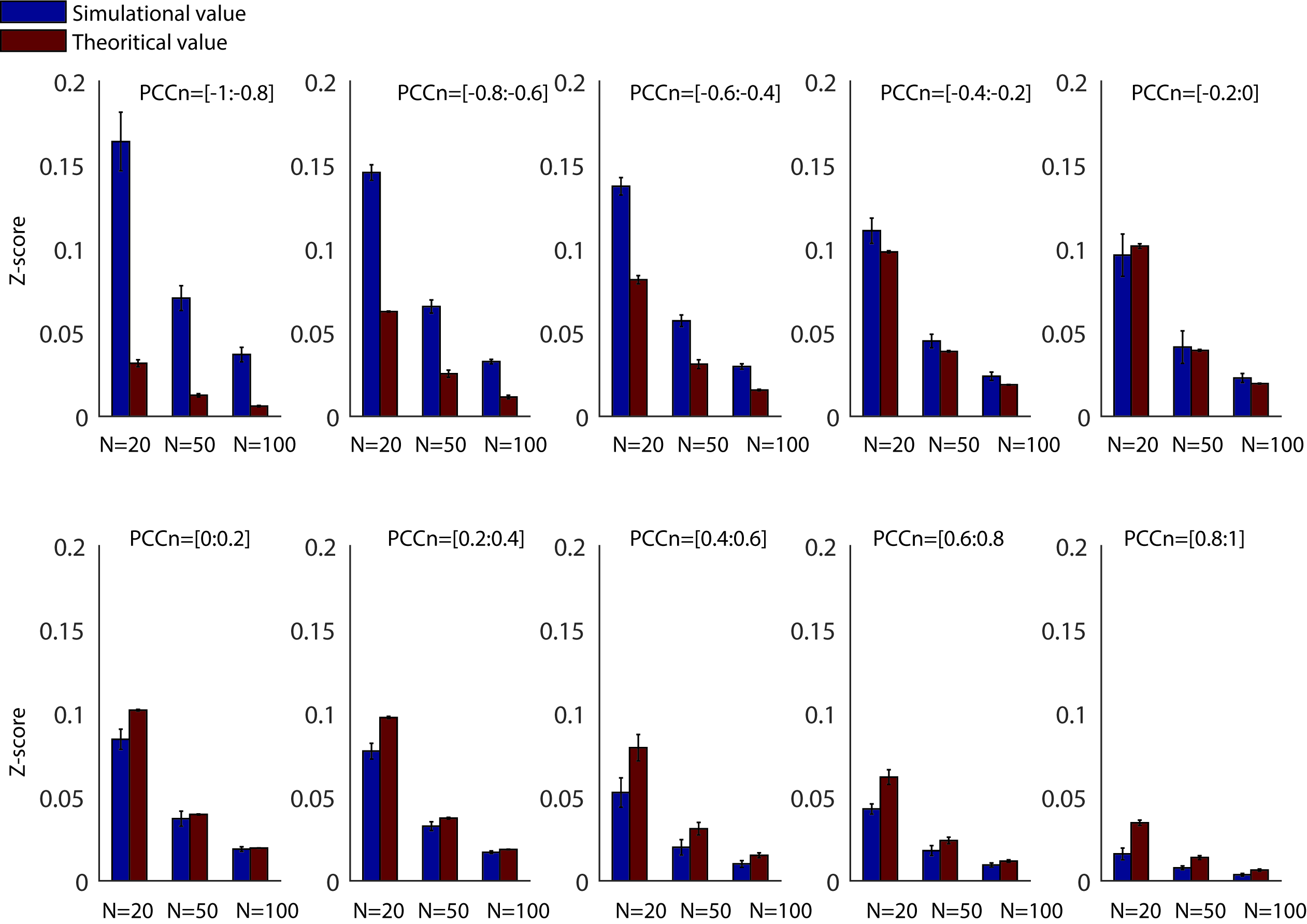


Fig S7 The significant z-score of Δ*PCCn* evaluated from the distribution of the random simulation (blue color) and the theoretical distribution (red color) by selecting randomly a value from ten uniform intervals from PCCn=[-1:1].


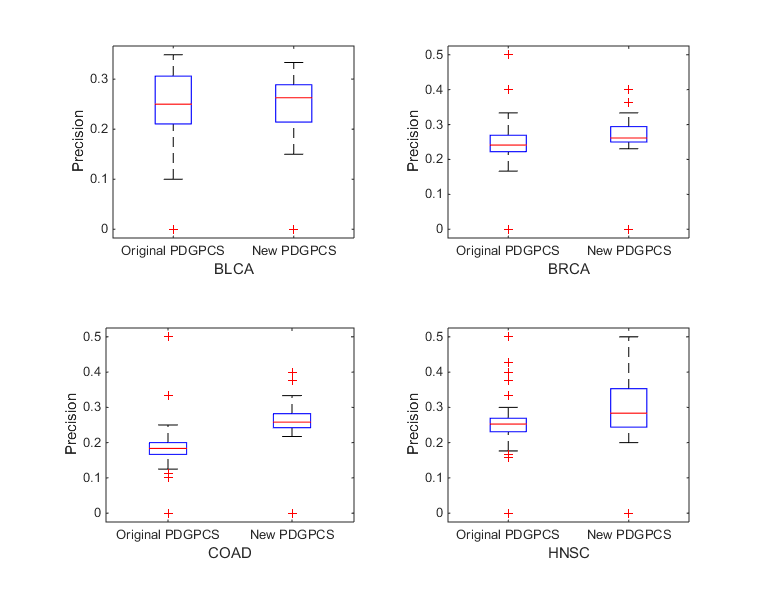


Fig S8 The boxplot of precision among top *k* (*k*=1,2,3,...50) ranking driver genes of original PDGPCS and new PDGPCS with deleting the edges with PCCn<-0.4 in BLCA, BRCA, COAD and HNSC cancer data sets.


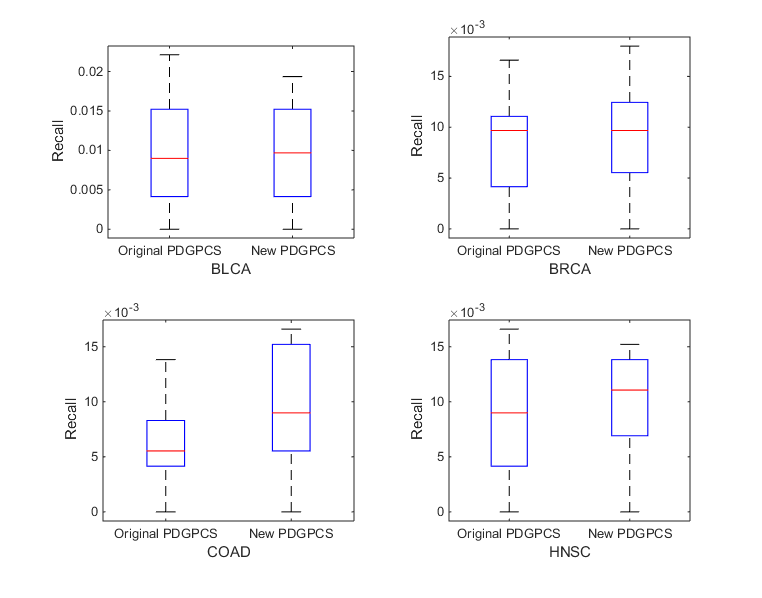


Fig S9 The boxplot of recall among top *k* (*k*=1,2,3,...50) ranking driver genes of original PDGPCS and new PDGPCS with deleting the edges with PCCn<-0.4 in BLCA, BRCA, COAD and HNSC cancer data sets.


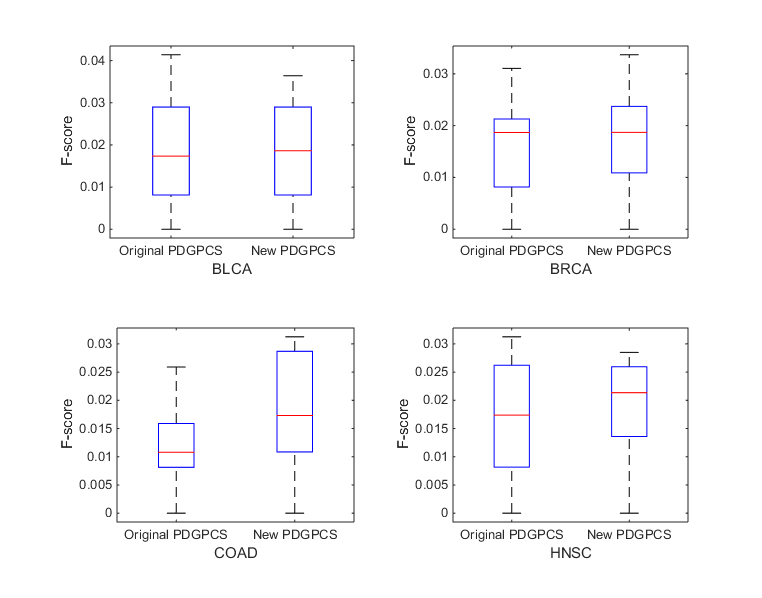


Fig S10 The boxplot of F-score among top *k* (*k*=1,2,3,...50) ranking driver genes of original PDGPCS and new PDGPCS with deleting the edges with PCCn<-0.4 in BLCA, BRCA, COAD and HNSC cancer data sets.


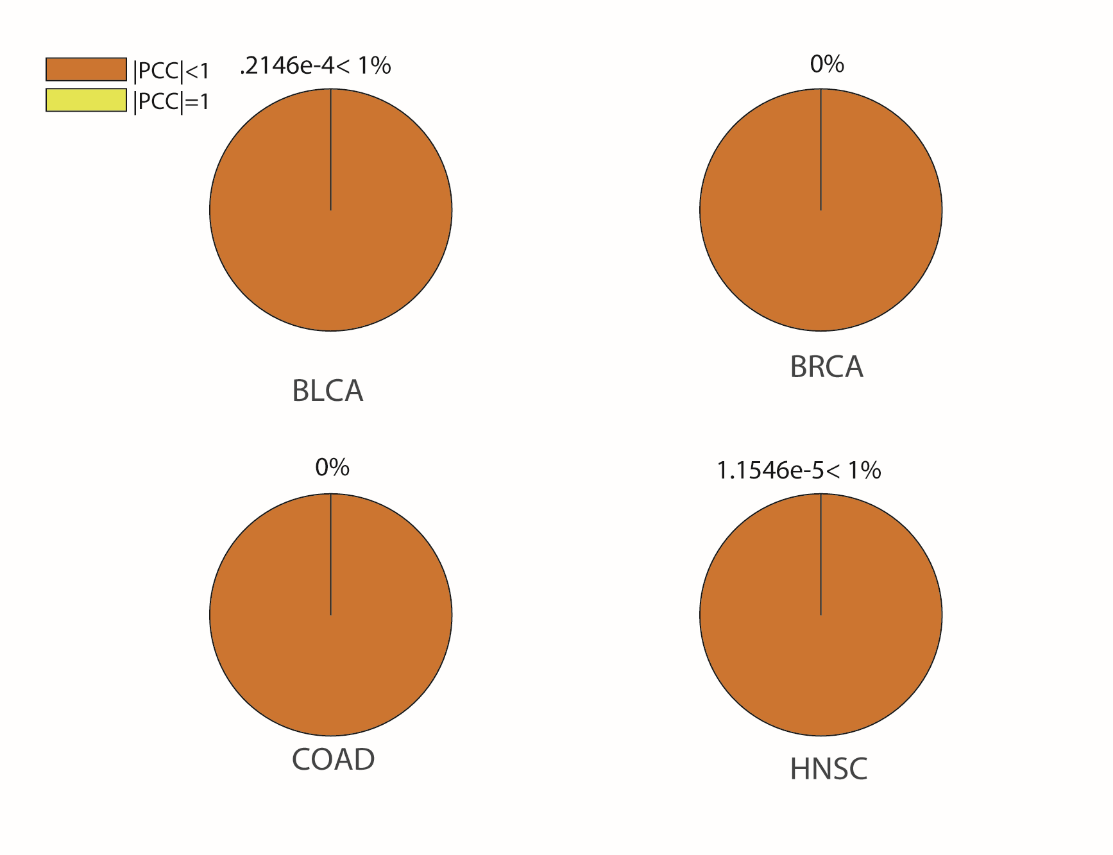


Fig. S11 The fraction of edges whose pearson correlation in the reference gene interaction network is +1 or -1 in BLCA, BRCA, COAD and HNSC cancer data sets.


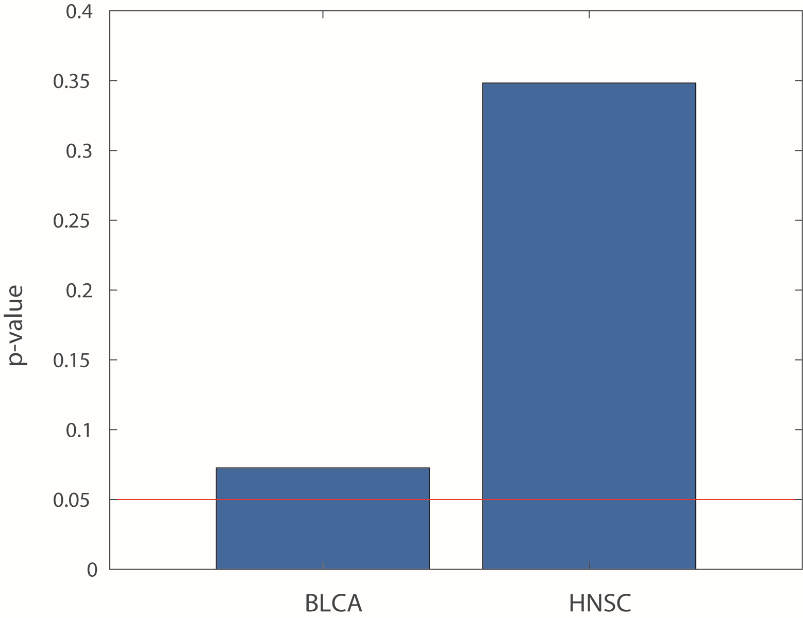


Fig. S12 The p-value of genes related with edges with *PCCn*=1 or -1 for enriching in CGC for BLCA and HNSC cancer data sets.


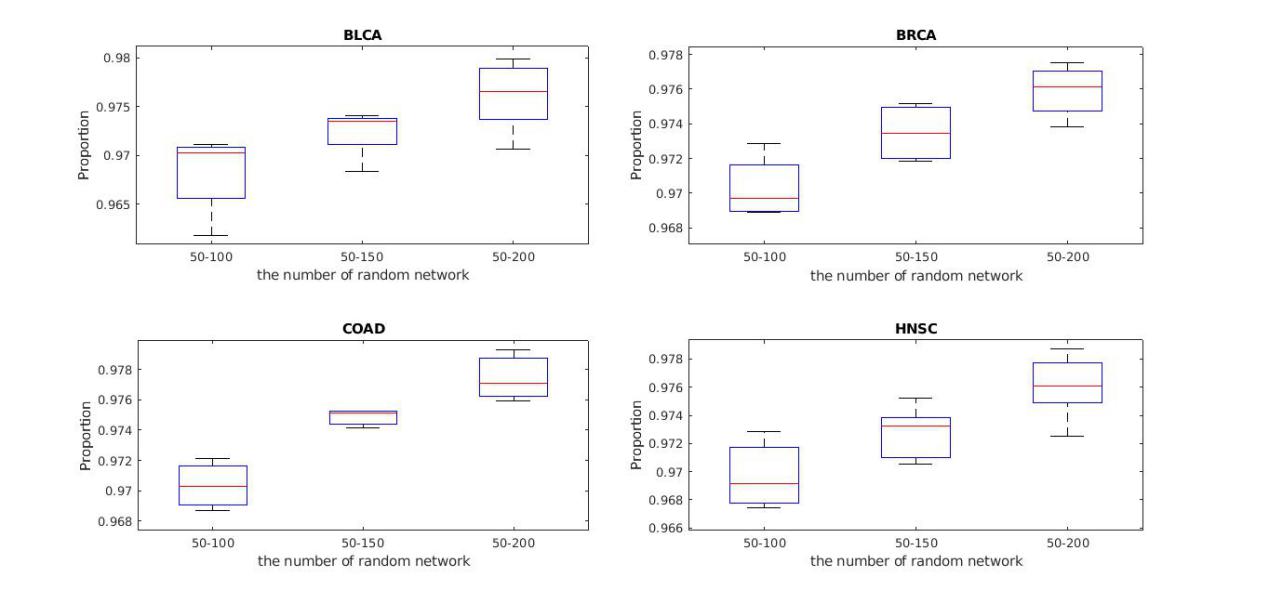


Fig. S13 The proportion of mutant genes with p-value>0.05 for 100, 150,200 random networks when taking 50 random networks as a reference.

Fig. S14 The explanation of the method (PCST) for producing a optimized steiner tree in a simple network.

Fig. S15 The explanation of the method (PCST) for not producing a optimized steiner tree in a simple network.
